# Loss of Twist impairs tentacle development and induces epithelial neoplasia in the sea anemone *Nematostella vectensis*

**DOI:** 10.1242/dev.205340

**Published:** 2026-08-20

**Authors:** Patricio Ferrer Murguia, Julia Hagauer, Emmanuel Haillot, Aissam Ikmi, Juan D. Montenegro, Alison G. Cole, Ulrich Technau

**Affiliations:** ^1^ Department of Neurosciences and Developmental Biology, Faculty of Life Sciences, University of Vienna, Djerassiplatz 1, 1030, Vienna, Austria; ^2^ Developmental Biology Unit, European Molecular Biology Laboratory, 69117 Heidelberg, Germany; ^3^ Research platform ‘Single cell regulation of stem cells’, University of Vienna, Djerassiplatz 1, 1030 Vienna, Austria; ^4^ Max Perutz labs, University of Vienna, Dr. Bohrgasse 5, 1030 Vienna, Austria

**Keywords:** Cnidaria, BHLH transcription factor Twist, Tentacles, Morphogenesis, FGF signalling, Appendages

## Abstract

Appendages are key features of the body plans of arthropods and vertebrates, yet little is known about appendage formation outside of bilaterians. Here, we investigate the molecular regulation underlying the formation of tentacles, the appendages in cnidarians. We find that the bHLH transcription factor Twist plays a crucial role in tentacle morphogenesis and tissue homeostasis in the sea anemone *Nematostella vectensis*. Using CRISPR/Cas9, we show that *twist* mutants exhibit impaired secondary tentacle formation, delayed and reduced proliferation in budding tentacles, and the absence of microcnemes, structures that demarcate tentacle boundaries, suggesting defects in spatial patterning. Loss of Twist disrupts expression of co-expressed mesodermal transcription factors *paraxis* and *tbx15* and perturbs the TOR-FGF signalling feedback loop necessary for normal tentacle growth. We note that the link of Twist and FGF signalling is reminiscent of limb formation in vertebrates. Moreover, starting at juveniles, mutants develop neoplasm-like epithelial overgrowth with tentacle-like molecular profiles, indicating a role for Twist in maintaining tissue homeostasis at the oral pole. Together, Twist integrates major signalling pathways to regulate secondary tentacle patterning and maintain spatial tissue organisation in the diploblastic *N. vectensis*.

## Teaser

**Summary:** The conserved transcription factor Twist is required for proper appendage formation in the sea anemone *Nematostella* through regulation of FGFR and TOR signalling.

## INTRODUCTION

**Fig. 1.**
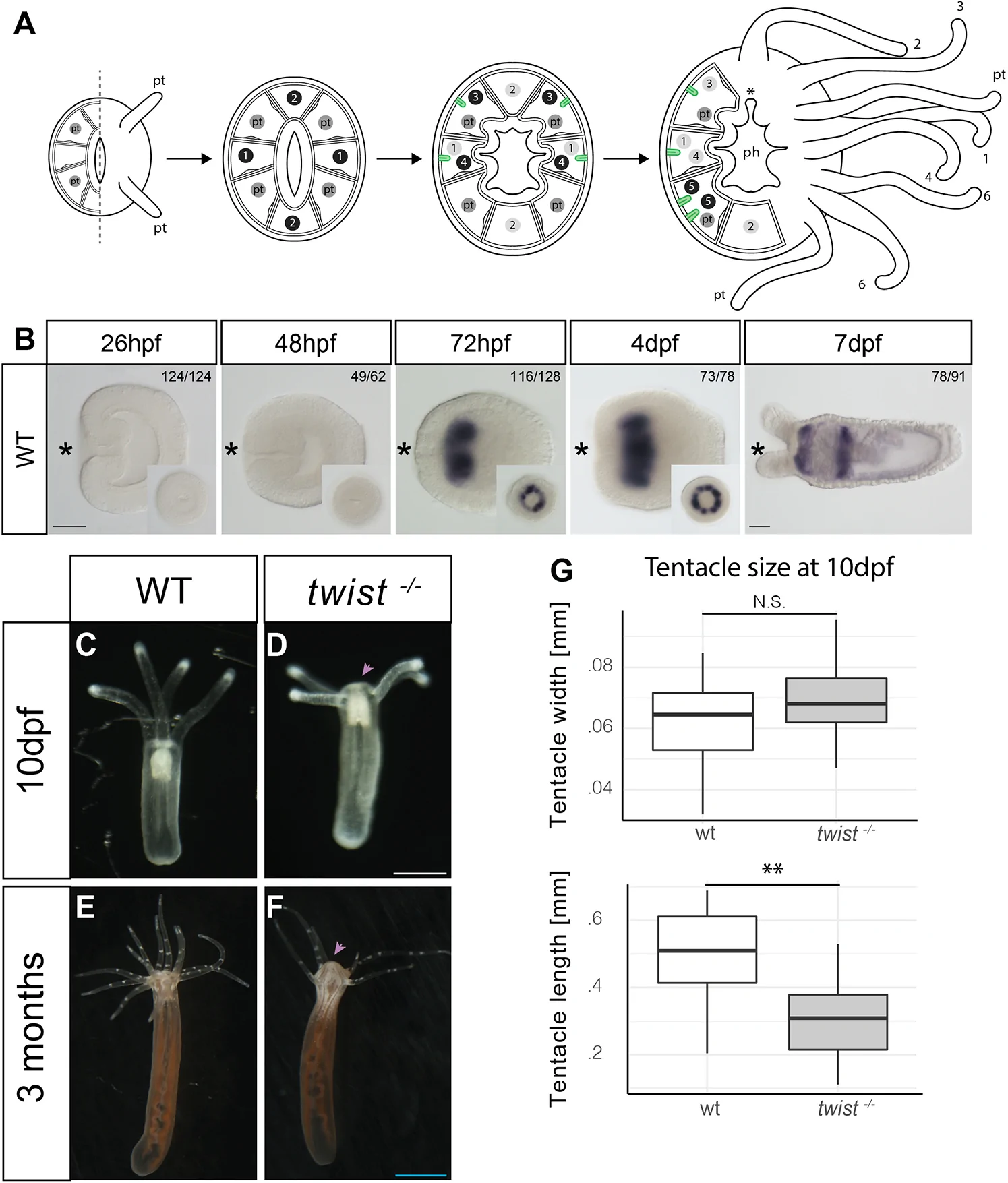
***twist*^−/−^ early mutant phenotype in *N. vectensis*.** (A) Schematic overview of tentacle development. Tentacles arise at the oral pole in a stereotyped sequence, with primary tentacles forming first and secondary tentacles added sequentially during segment subdivision. Numbered black circles indicate the order of tentacle addition. The pharynx (ph), primary tentacles (pt), siphonoglyph (*) and mesenteries are shown for orientation. Microcnemes are shown in green. (B) Whole-mount *in situ* hybridisation showing *twist* expression in wild-type (WT) animals. Asterisk indicates the oral pole. (C,D) WT (C) and *twist*^−/−^ (D) primary polyps at 10 dpf. Mutants show a protruding oral disc (pink arrow) instead of the flat mouth observed in WT. (E,F) WT (E) and *twist*^−/−^ (F) polyps at 3 months, with persistent oral disc defects in mutants (pink arrow). (G) Quantification of tentacle width and length in WT (*n*=30) and *twist*^−/−^ mutants (*n*=29) at 10 dpf. Tentacles are significantly shorter in mutants, while width is unchanged. **P<2.16×10^−6^ (two-sided Wilcoxon rank-sum test). N.S., not significant. Box plots show median values (middle bars) and first to third interquartile ranges (boxes); whiskers indicate 1.5× the interquartile ranges. Scale bars: 50 μm (B); 500 μm (C,D); 5 mm (E,F).

Numerous animals have appendages, such as legs, fins, antennae and tentacles, that support locomotion, feeding and sensory perception (Heming, 1996; Panganiban et al., 1997; Abzhanov et al., 2001; Fritz et al., 2013). Appendage development has been best studied in *Drosophila* and vertebrates. In insects, patterning of wings and legs arises at the intersection of Wnt, BMP and Hedgehog signalling domains, which define the centre of the proximodistal axis and subsequently activate transcriptional programmes including the homeobox transcription factor Distal-less (*Dll*) (reviewed by Ruiz-Losada et al., 2018). In vertebrates, limb formation is initiated by the local mesodermal expression of Tbx5 or Tbx4 in the forelimb and hindlimb, respectively, in conjunction with the basic Helix-Loop-Helix (bHLH) transcription factor Twist (O'Rourke and Tam, 2002; Zúñiga et al., 2002). This induces the expression of FGF10 and FGFR in the mesoderm and, indirectly, FGF8 in the overlying ectoderm, the apical ectodermal ridge (AER) (reviewed by McQueen and Towers, 2020). The distinct developmental regulation of appendage formation in insects and vertebrates raises the question of whether appendage formation has a common evolutionary origin in bilaterians. To address this question, the outgroup of bilaterians, cnidarians, are crucial. Cnidarian tentacles are such appendages, and the tentacles are mostly used to catch prey, sense the environment and act in defence. They can vary in morphology and number, but all share the common feature of having a high density of stinging cells, the phylum-defining cell types (Zenkert et al., 2011; Fritz et al., 2013; Gold et al., 2015; Fujiki et al., 2019). What controls the formation of the tentacles in a highly defined spatio-temporal pattern remains largely unsolved. The starlet sea anemone *Nematostella vectensis* has become a well-established model organism due to its ease of laboratory maintenance, reliable spawning and robust molecular tools (Hand and Uhlinger, 1992; Genikhovich and Technau, 2009a). In *N. vectensis*, the tentacles form at the oral pole as extensions of the body wall, surrounding the mouth. The polyp has eight mesenteries that form during larval development. They divide the animal into eight segments, which serve as initial boundaries between neighbouring tentacles (Ryan et al., 2007; He et al., 2018). The onset of tentacle development and extension is marked by changes in the ectoderm forming a placode, followed by modifications in the epithelial cell layer that drive elongation (Fritz et al., 2013). Four tentacle anlage are formed during larval development, and they form the first four tentacles present in the primary polyp. They are positioned in every other segment as defined by the mesenteries. The next four tentacles form in the remaining segments between the primary tentacles. Later, in a feeding-dependent manner and through several stereotyped phases, secondary tentacles are added, commonly resulting in a total of sixteen tentacles (Ikmi et al., 2020). To achieve this, each segment must be divided through the formation of microcnemes, which appear as incomplete mesenteries and act to delineate boundaries between neighbouring tentacles (Fig. 1A).

Some of the molecular mechanisms for normal tentacle development in *N. vectensis* have already been described. Specifically, in developing larvae, a set of Hox genes, together with *gbx*, are expressed in the gastric tissue of the planula larva in a staggered manner along the directive axis, which is perpendicular to the main oral-aboral axis. Their expression boundaries mark precisely the position of the later mesenteries (He et al., 2018). All hox genes and *gbx* are regulated by a BMP signalling gradient along the directive axis (Saina et al., 2009; Genikhovich et al., 2015) and delineate the segments necessary for initial tentacle development and patterning (Finnerty et al., 2004; Ryan et al., 2007; He et al., 2018). In a recent study, FGF signalling was found to play a role in tentacle formation. Specifically, a crosstalk between Fibroblast Growth Factor Receptor b (FGFRb) and Target of Rapamycin (TOR) in the gastrodermal layer drives the development of secondary tentacle growth (Ikmi et al., 2020). At present it is unknown what regulates the local activation of the FGFR expression in the early tentacle bud anlage. In some bilaterian species, the bHLH transcription factor Twist acts upstream of FGFR signalling, regulating various developmental processes, including the growth and extension of appendages (Hornik et al., 2004; Lu et al., 2012; Zhu et al., 2014). Notably, *Nematostella twist* is expressed in the mesodermal lining of the pharynx and at lower levels in the body wall (Martindale et al., 2004), the region in which TOR signalling activates FGFR signalling during secondary tentacle formation. However, the upstream regulation of *twist* and its role in tentacle morphogenesis in *Nematostella* remain unexplored.

Here, we report that *twist* is required for proper formation of secondary tentacles through regulation of the TOR signalling pathway. We further show that *twist* expression is regulated by Wnt, Notch and BMP signalling, while MAPK and Hedgehog inhibition do not affect its expression. Transcriptomic analysis of *twist* mutants reveals a coordinated downregulation of mesoderm-associated transcription factors alongside changes in major developmental signalling pathways, including Wnt, Hedgehog and FGF. Moreover, in juvenile to subadult stages of the polyp, *twist* mutants form amorphic hyperplasia of the body wall with tentacle tissue identity, suggesting that Twist might be necessary for localised and directed outgrowth of the tentacle appendages. These results indicate that Twist functions at the intersection of major developmental signalling pathways and a mesodermal-like transcriptional programme in *Nematostella*.

## RESULTS

### *twist^−/−^* mutants develop eight mesenteries and shorter primary tentacles

Expression analyses using *in situ* hybridisation confirmed that *twist* expression is not detectable during early gastrulation (Fig. 1B), and only faintly expressed in the 2-day-old planula (Fig. 1B). By 72 h post-fertilisation (hpf) expression becomes clearly detectable in the inner layer of the pharynx (Martindale et al., 2004; Fig. 1B). Consistent with recent work assigning mesoderm-like identity to the cnidarian inner layer and endodermal features to pharyngeal ectoderm, we use ‘mesoderm’ when referring to the inner gastrodermis in *Nematostella* (Steinmetz et al., 2017; Haillot et al., 2025). Expression is maintained throughout the late planula and polyp stages, including post-feeding juvenile polyps, where *twist* remains detectable in the mesodermal lining of the pharynx, consistent with earlier stages (Fig. 1B). Expression was primarily observed in the mesenterial chambers. Notably, in the polyps, the expression domain appeared to be split into two distinct regions: one at the outer pharynx below the tentacles and one in the lower, inner pharynx (Fig. 1B). As the animal transitions into the polyp, *twist* expression adopted a ‘cage’-like pattern around the pharynx, being more prominent at the bottom of the pharynx and around the primary tentacles.

To study the function of the transcription factor Twist in the sea anemone *N. vectensis* we generated a *twist* knockout using CRISPR/Cas9 (for details see Materials and Methods). The 5 bp deletion induced a frameshift after the first helix of the bHLH domain, introducing a premature stop codon after 67 amino acids. The truncation removes the loop and second helix of the bHLH domain and the protein is thus unable to form dimers (Fig. S1A-C). To assess structural changes, we predicted the tertiary structure of the wild-type (WT) and Twist mutant domains with AlphaFold (Jumper et al., 2021) and found that the mutant lacks the canonical bHLH fold (Fig. S1C). Interestingly, the homozygous *twist* mutant still shows the same expression pattern as the WT, suggesting that nonsense-mediated decay may not be active in *Nematostella* (Fig. S1D).

Although early expression of *twist* is detected at the planula stage, *twist^−/−^* mutants form all eight mesenterial segments with preserved overall organisation, and no major defects in segment number or arrangement were observed (Fig. S2; Movies 1,2). Notably, at 10 days post-fertilisation (dpf), the first morphological differences between WT and *twist^−/−^* animals become apparent. The oral disc of *twist^−/−^* animals displayed a protrusion that becomes increasingly pronounced as animals matured, resembling a hypostome-like morphology (Fig. 1C-F; Fig. S3). However, overall body organisation, mesentery number and primary tentacle patterning remained preserved at early polyp stage. Additionally, the primary tentacles in WT animals were somewhat longer than those of *twist^−/−^* mutants at the same developmental stage (Fig. 1G).

### Twist expression is regulated by Wnt, Notch and BMP signalling and is required for mesodermal transcription networks in *Nematostella*

**Fig. 2.**
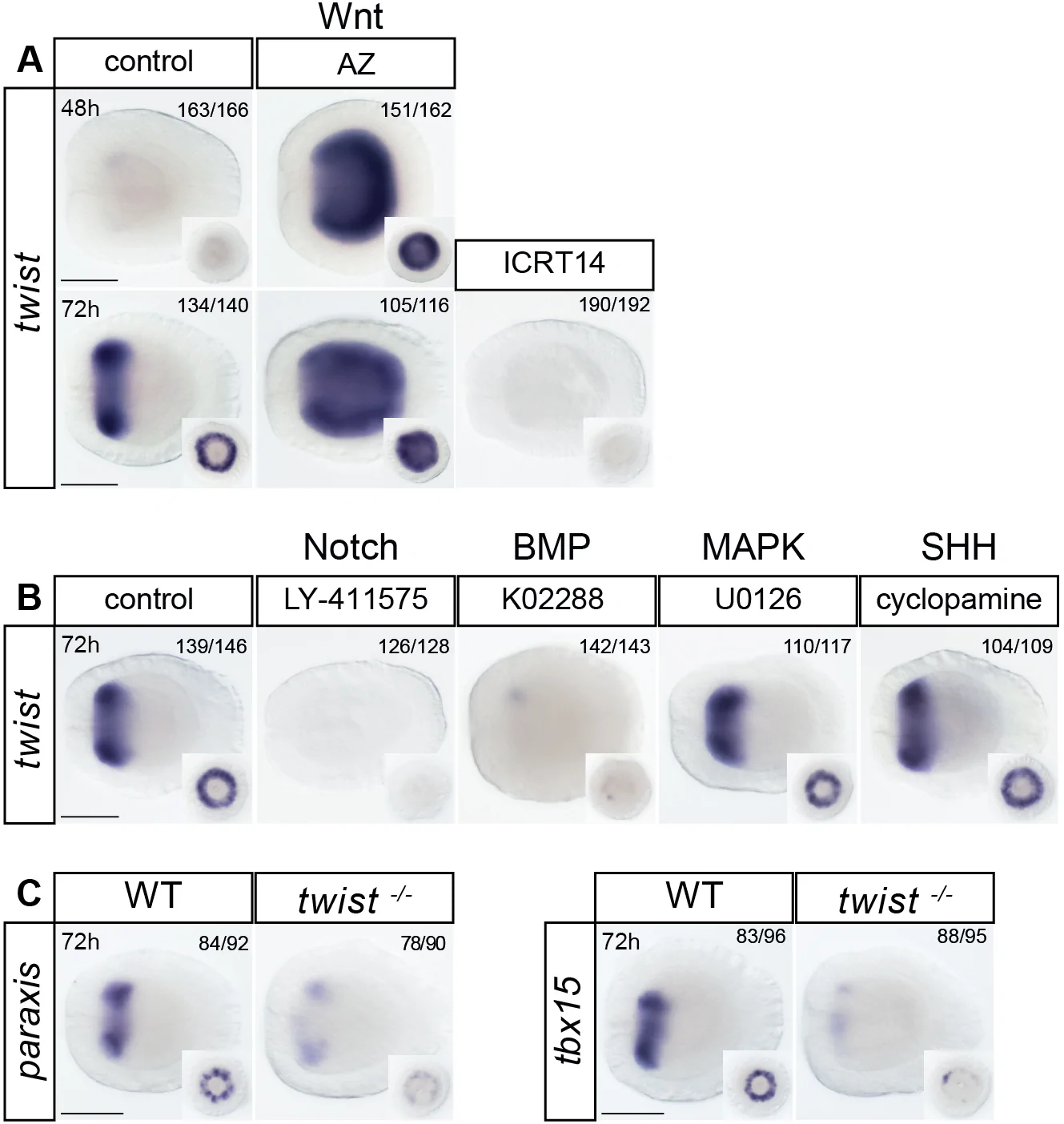
***twist* is regulated by Wnt, Notch and BMP signalling and required for *paraxis* and *tbx15* expression.** (A) *twist* expression at 48 h and 72 h following modulation of Wnt signalling. Activation with Azakenpaullone (AZ) expands *twist* expression, while inhibition with ICR14 abolishes expression. *n*=166 (control 48 h), 162 (Az 48 h), 140 (control 72 h), 116 (AZ 72 h), 192 (ICR14 72 h). (B) Inhibition of Notch with LY-411575 and of BMP with K02288 reduces or eliminates *twist* expression. Inhibition of MAPK with U0126 or of Hedgehog signaling with cyclopamine has no detectable effect. *n*=146 (control), 128 (LY-411575), 143 (K02288), 117 (U0126), 109 (cyclopamine). (C) Expression of *paraxis* and *tbx15* is reduced in *twist*^–/–^ embryos compared with wild type. *n*=92 (WT *paraxis*), 90 (*twist*^−/−^ *paraxis*), 96 (WT *tbx15*), 95 (*twist*^−/−^ *tbx15*) animals examined at 72 h. Lateral views; oral views shown as insets. Controls were all in DMSO. All panels show biological replicates from a single experiment representative of three independent experiments with similar results. Scale bars: 50 μm.

To determine how *twist* expression is regulated, we pharmacologically inhibited key signalling pathways (predominantly 48-72 hpf) and analysed *twist* expression using *in situ* hybridisation (Fig. 2A,B). Our results revealed that *twist* expression is regulated by Wnt, BMP and Notch signalling but is independent of MAPK and Hedgehog signalling. Ectopic activation of the Wnt/β-catenin pathway led to an expansion of *twist* expression throughout the whole mesoderm (Fig. 2A). Conversely, inhibition of Wnt signalling completely abolished *twist* expression (Fig. 2A). Additionally, both Notch and BMP signalling are required for normal *twist* expression. Notch inhibition resulted in a total loss of *twist* expression, while BMP signalling inhibition significantly reduced or abolished *twist* (Fig. 2B). In contrast, inhibition of MAPK or Hedgehog signalling had no effect, indicating that these pathways do not regulate *twist* expression in *Nematostella* (Fig. 2B). To further explore the molecular consequences of the complete loss of Twist in the planula stage, we examined the expression of two co-expressed transcription factors, *paraxis* and *tbx15*, in the *twist^−/−^* mutants. Both transcription factors showed strong downregulation in the mutant when compared with the WT (Fig. 2C), indicating that Twist is upstream and necessary for the expression and/or maintenance of *paraxis* and *tbx15*. This places *twist* upstream within the pharyngeal mesodermal transcriptional regulatory network in *Nematostella*.

### Secondary tentacle formation was severely delayed or abolished in the *twist* mutant

**Fig. 3.**
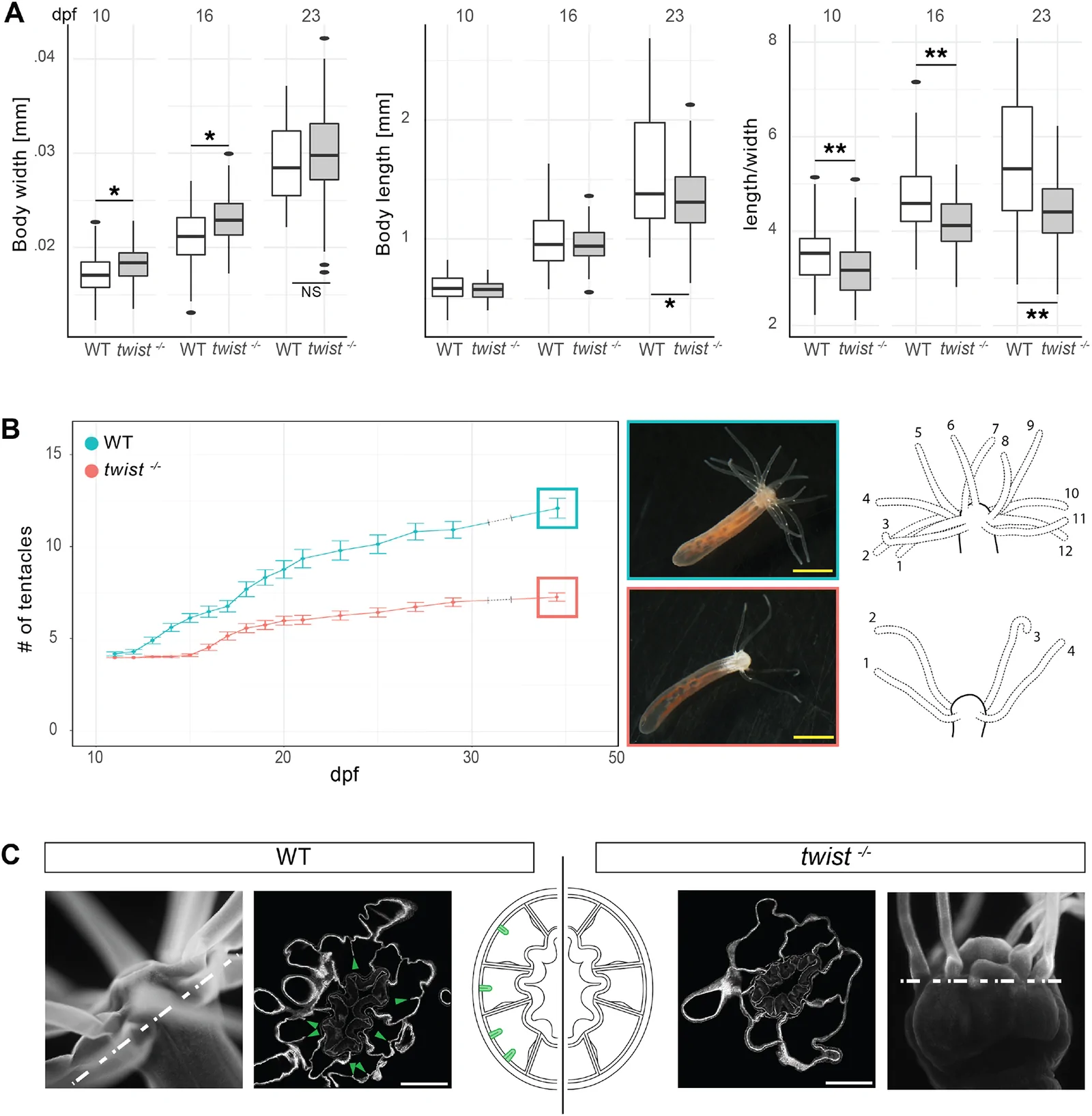
***Twist* mutants show altered body proportions, impaired tentacle development, and lack oral microcnemes.** (A) Quantification of body width (left), length (centre) and width-to-length ratio (right) in wild-type (WT) and *twist^−/−^* polyps. *n*=20 juvenile polyps per genotype. Statistical comparisons by two-tailed Welch's *t*-test. Width: *P=8.8×10^−5^ (dpf 10), *P=9.1×10^−8^ (dpf 16), *P*=0.24 (dpf 23). Length: *P*=0.11 (dpf 10), *P*=0.21 (dpf 16), *P=0.0097 (dpf 23). Length/width ratio: **P=1.5×10^−4^ (dpf 10), **P=3.5×10^−8^ (dpf 16), **P=8.4×10^−5^ (dpf 23). NS, not significant. Box plots show median values (middle bars) and first to third interquartile ranges (boxes); whiskers indicate 1.5× the interquartile ranges; dots indicate outliers. (B) Tentacle number in WT (cyan) and *twist^−/−^* (red) animals across 46 dpf. Representative images show a juvenile WT polyp with 12 tentacles and a *twist^−/−^* mutant with only four at 46 dpf. Sketches next to the representative images indicate the arrangement and tentacle count for WT and *twist^−/−^* polyps at 46 dpf. *n*=20 juvenile polyps per genotype. Single experiment. (C) Confocal cross-sections of adult polyps at the oral pole stained with Phalloidin (grey). WT animals show distinct oral microcnemes (green arrowheads), absent in *twist^−/−^* mutants. Central schematic illustrates oral pole organisation in WT and *twist^−/−^* animals, highlighting the presence/absence of oral microcnemes at the base of the body wall. *n*=5 WT and *n*=5 *twist*^−/−^ adult animals. Dashed line indicates a sagittal section. Scale bars: 5 mm (B); 100 μm (C).

At roughly 14-15 dpf, the formation of secondary tentacles begins, which is promoted by feeding (Ikmi et al., 2020). To ascertain whether secondary tentacle development was hampered, we started to feed animals every day from 10 dpf. To make sure that *twist* mutants and WT were feeding properly, the animals' growth (length and width) was also monitored. No initial differences in body length were observed: only after 13 days of constant feeding did the *twist^−/−^* animals become somewhat shorter than the WT polyps, with an average reduction in length of 15.5%. WT polyps were initially significantly narrower than mutant polyps at days 0 and 6 post-feeding (*P*=8.8×10^−5^ and *P*=9.1×10^−8^, respectively; Welch's *t*-test), with no significant difference at day 13 (*P*=0.24). The body length to width ratio of the mutant is considerably lower at all recorded time intervals, showing that mutant polyps of the same length are frequently broader (Fig. 3A).

**Fig. 4.**
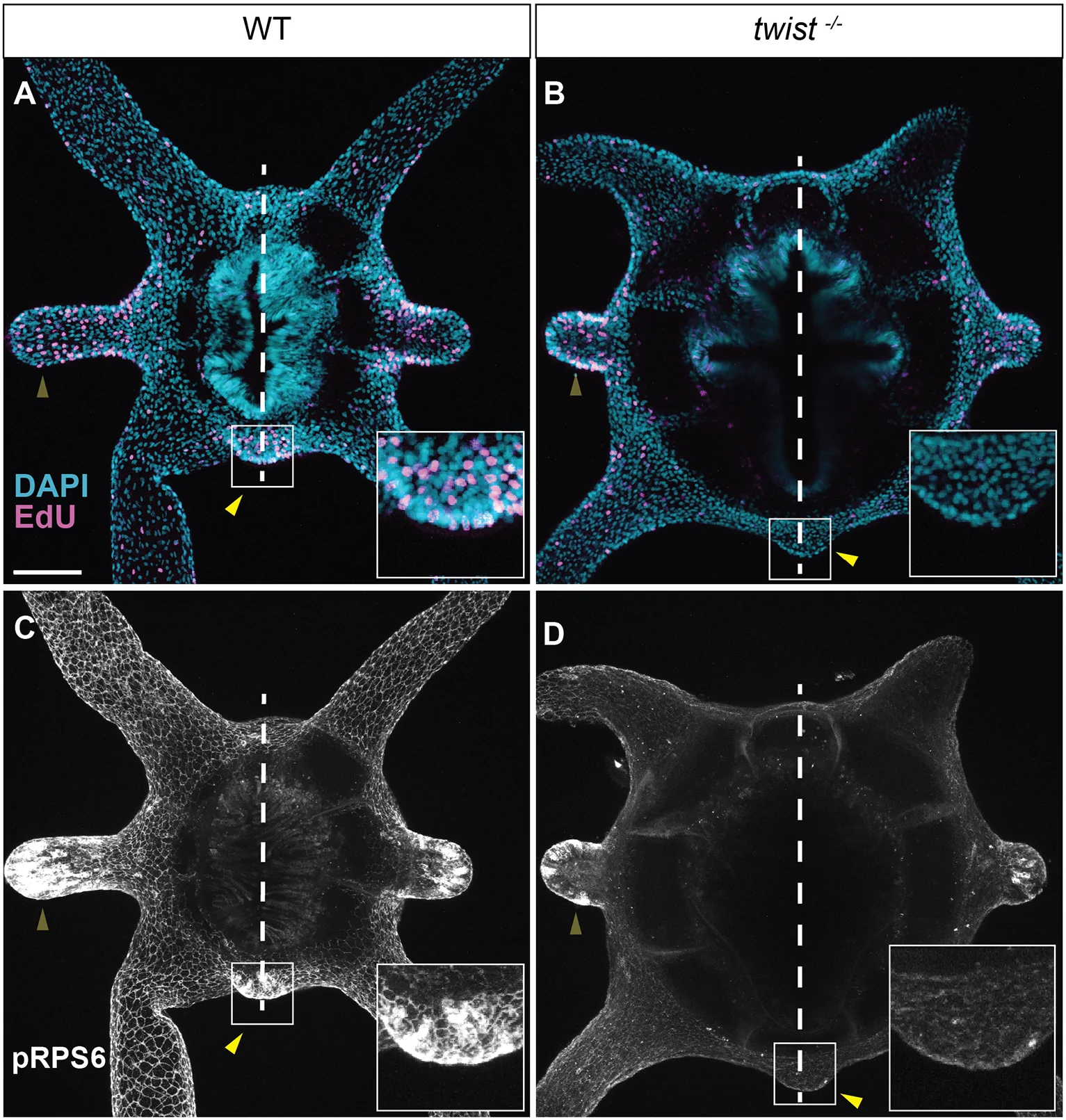
**Tentacle bud development and TOR activity is impaired in *twist* mutants.** (A) EdU staining of an overhead view of a wild-type (WT) polyp 3 days post feeding (13 dpf). (B) EdU staining of an overhead view of a *twist* mutant polyp 6 days post feeding (16 dpf). (C) Immunostaining for pRPS6, indicating TOR pathway activity, in a WT polyp. (D) Immunostaining for pRPS6 in a *twist* mutant polyp. Yellow arrowheads indicate early tentacle buds; faded yellow arrowheads indicate secondary tentacles that are already elongating. *n*=4 animals per genotype, each from an independent experiment. All panels show representative individuals. Dashed lines indicate the directive axis. Scale bar: 100 μm.

The most striking phenotype of the *twist^−/−^* mutants at the juvenile polyp stage is found in the formation of secondary tentacles. Over the 46-day observation period, WT animals typically reached up to 12 tentacles (as expected at this juvenile stage), before they reached their final number of 16 tentacles at the adult stage. By contrast, *twist^−/−^* mutants generally developed only 4-8, in rare cases a maximum of 10 tentacles (Fig. 3B). Some mutants retained only the four primary tentacles, without ever fully developing secondary tentacles (Fig. 4A,B). Moreover, if secondary tentacles were formed, they were significantly thinner and shorter in *twist^−/−^* mutants (Fig. 3B; Fig. S3). Later phases of secondary tentacle addition were typically followed by pseudo-segmentation of the eight body chambers, known as microcnemes. We therefore examined cross-sections of adults for these mesodermal folds. Interestingly, while microcnemes were present in all WT animals, they were completely absent in the mutants, with this phenotype being fully penetrant across all animals analysed (*n*=5 WT, *n*=5 *twist^−/−^*) (Fig. 3C).

In WT animals, the outgrowth of new secondary tentacles is preceded by local spots of proliferation (Ikmi et al., 2020). We therefore performed EdU pulse labelling on growing tentacle buds. While the early and elongating tentacle buds of WT polyps showed localised proliferation as reported before (Ikmi et al., 2020), the early buds of mutant polyps did not exhibit any increased proliferation (Fig. 3A,B). We next investigated the TOR and FGF pathways in the *twist^−/−^* mutants. We used immunostaining to detect the presence of phosphorylated ribosomal protein S6 (pRPS6), a marker of TOR activity that has been shown to play a role between nutrient uptake and signalling processes in the development of secondary tentacles (Ikmi et al., 2020). In contrast to WT animals, in which pRPS6 is detected in both initial and extending tentacle buds, pRPS6 was exclusively detected in extending tentacle buds in the *twist*^−/−^ mutant (*n*=4 per genotype) (Fig. 4C,D).

**Fig. 5.**
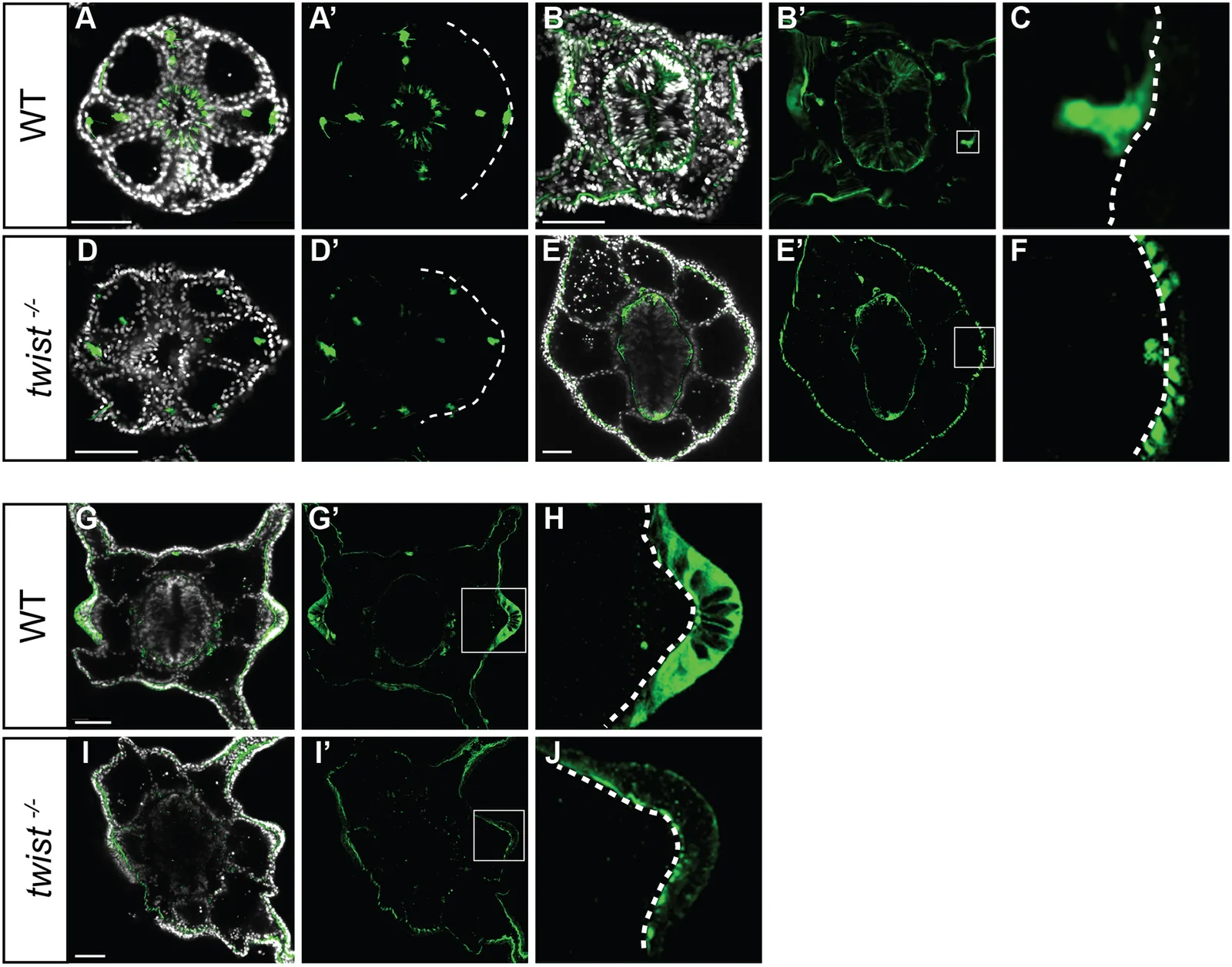
***Fgfrb-eGFP* expression in budding tentacles is reduced in *twist^−/−^* animals.** (A,A′,D,D′) Fgfrb-eGFP expression in ring muscles of 10 dpf unfed wild-type (WT) (A,A′) and *twist^−/−^* (D,D′) primary polyps; DAPI staining (white) shown in A,D. (B,B′,E,E′) Fgfrb-eGFP expression in WT (B,B′) and *twist^−/−^* (E,E′) animals 4 days after feeding (14 dpf); DAPI staining (white) shown in B,E. Reduced signal seen in *twist^−/−^* animals. (C,F) Close-up of Fgfrb-eGFP expression in WT (C) and *twist^−/−^* (F) ring muscles 4 days after feeding. (G,G′,I,I′) Fgfrb-eGFP expression in secondary tentacle buds of WT (15 dpf; G,G′) and *twist^−/−^* (18 dpf; I,I′) animals; DAPI staining (white) shown in G,I. (H,J) Close-up of Fgfrb-eGFP expression in WT (H) and *twist^−/−^* (J) secondary tentacle buds. Reduced signal seen in *twist^−/−^* secondary tentacle buds. Dashed line marks the boundary between mesoderm and ectoderm. *n*=5 animals per genotype in total, from three independent experiments. All panels show representative individuals. Scale bars: 100 μm.

The spot-like expression domain of FGFRb expression at the site of the future tentacle outgrowth could be visualised in a FGFRb-eGFP reporter line in the WT animals (Ikmi et al., 2020). To further explore the role of Twist in the regulation of FGF signalling, we generated a *twist^−/−^*/Fgfrb-eGFP mutant transgenic line to examine any changes in the Fgfrb-positive ring muscle cells in both fed and unfed polyps. In unfed primary polyps, Fgfrb-eGFP-positive cells were detectable in both WT and *twist^−/−^* animals, although signal intensity and distribution were more variable and in some cases slightly reduced in *twist^−/−^* mutants (Fig. 5A,A′,D,D′). Upon feeding and maturation, the Fgfrb-eGFP-positive cells in *twist^−/−^* mutants became consistently weaker and smaller, though not completely abolished (Fig. 5B,B′,C,E,E′,F). Furthermore, as the secondary tentacles started to elongate, the Fgfrb-eGFP-positive cells in *twist^−/−^* mutants became significantly weaker compared to the WT (Fig. 5G,G′H,I,I′,J). Our observations suggest that Twist plays a role in maintaining the signalling dynamics between the TOR and FGF pathways, which are essential for proper tentacle growth.

Since secondary tentacles in the *twist^−/−^* mutants do not develop normally, we wondered whether the structure and composition of the tentacles is normal. First, we assessed the tentacle retractor muscle. Most muscles in *Nematostella* are located in the gastrodermis, with the exception of the tentacle retractor muscles, which are of ectodermal origin (Jahnel et al., 2014; Cole et al., 2023). The tentacle ectodermal retractor muscles are longitudinally orientated, whereas gastrodermal muscles are circular. We found that muscle organisation was maintained even if the secondary tentacles in *twist^−/−^* mutants were shorter and often thinner than those found in WT animals (Fig. S4A,A′,B,B′). We conclude that *twist* has no role in muscle formation of the tentacles, as in the rest of the body. Secondly, we checked whether the three cnidocyte types (basitrichous haplonema, microbasic mastigophore and spirocytes) that are typically found in WT tentacles were also present in the mutant tentacles. Late basitrichous haplonemas and microbasic mastigophores were detected with high DAPI concentrations (Fig. S4C,C′). An antibody against the minicollagen NvNcol-3 (kindly provided by the Holstein laboratory, University of Heidelberg), which recognises all three different cell types, was used to mark early developing nematocytes (Fig. S4D,D′). In parallel, we dissociated individual tentacles and, using differential interference contrast (DIC) microscopy, we assessed all three types of nematocytes by morphology. All types were present in the *twist^−/−^* and WT tentacles, and there were no apparent changes in cnidocyte development in the mutant (Fig. S4E-G′). This suggests that *twist* has no direct role in cnidocyte development. Lastly, we generated single cell transcriptomes from *twist^−/−^* tentacles and compared them with tentacle WT libraries. The analyses suggested that the *twist*^−/−^ mutants do not have any missing cell types. When comparing the relative abundance of each cell type, normalised to the total of cells per sample, we observed a decrease in spirocytes, muscle cells, neurons and mucus glands. Yet, the reduction in muscle cells may reflect the smaller tentacle size observed in *twist*^−/−^ mutants and could contribute to their altered morphology when compared to the WT (Fig. S5A,B).

### *twist^−/−^* mutants show aberrant oral disc morphology and display a neoplasm-like outgrowth

**Fig. 6.**
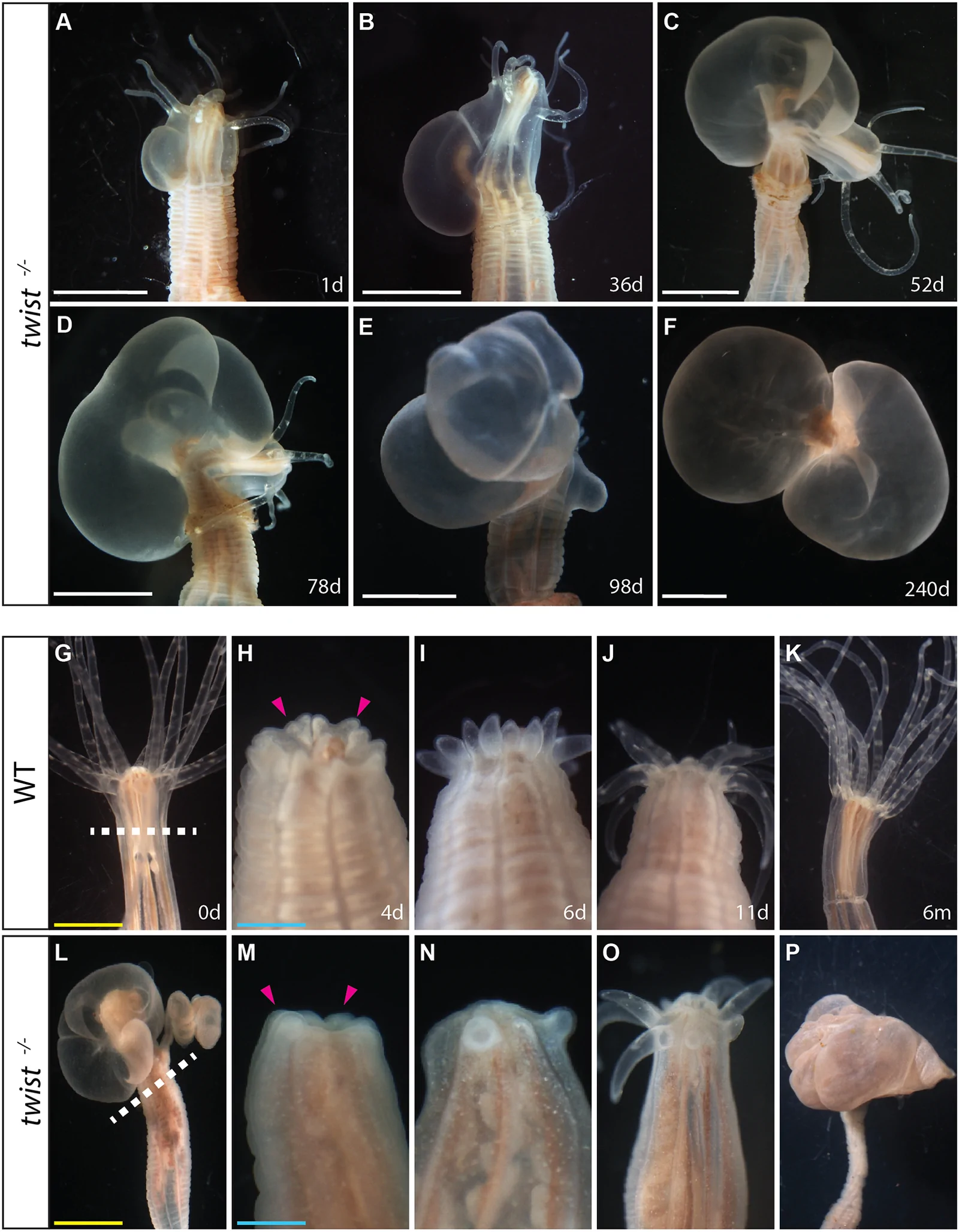
**Impaired tentacle regeneration and neoplasm-like formation in *twist^−/−^* mutants.** (A-F) Neoplasm-like growth documentation: day 1 (A), day 36 (B), day 52 (C), day 78 (D), day 98 (E) and day 240 (F). (G-P) Wild-type (WT) (G-K) and *twist^−/−^* (L-P) animals at various time points following oral amputation (cut indicated by dashed line in G and L). (H,M) 4 days post-amputation: magenta arrowheads mark the appearance of new tentacle buds. (I,N) 6 days post-amputation: WT animals display synchronous regrowth of all tentacles, whereas *twist^−/−^* animals show limited and asynchronous tentacle regeneration. (J,O) 10 days post-amputation: WT animals continue to regenerate tentacles uniformly, whereas *twist^−/−^* animals exhibit uneven tentacle development. (K,P) 6 months post-amputation: WT have regenerated all 16 tentacles, whereas *twist^−/−^* display a reappearance of a neoplasm-like. (L) Neoplasm-like is visible at the oral pole before amputation in twist^−/−^ animals. Panels A-F show a single representative *twist*^−/−^ animal documented over 240 days. Panels G-P show single representative animals per genotype following oral amputation. The neoplasm-like phenotype was observed in multiple independent *twist*^−/−^ animals across the course of the study. Scale bars: 5 mm (A-F); 3.5 mm (G,L); 2.5 mm (H-K,M-P).

The morphological differences between *twist*^−/−^ and WT animals became more pronounced as the animals grew. In the *twist^−/−^* mutants, primary tentacles got shorter and thinner, much like the secondary tentacles in mutants. In addition, merged secondary tentacles were frequently observed. This phenotype is possibly due to the absence of microcnemes in *twist*^−/−^ animals, which normally subdivide the eight mesenteries and establish clear tentacle boundaries. Additionally, in contrast to the essentially flat mouth ends of the WT, the initial light trapezoid-like structure seen in the primary polyp oral disc became more apparent in the adults (Fig. S3B). Most strikingly though, in *twist*^−/−^ mutants starting at the juvenile stage (about 1.5 months post-fertilisation), a bubble-shaped neoplasm-like epithelial structure began to stochastically appear on one side of the animal between two mesenteries (Fig. 6A-D). This expansion, which included both cell layers, was mostly found in the *twist*-expressing region of the body wall at the level of the pharynx, although it was occasionally seen to extend to lower sections of the animal. The growth of the epithelial neoplasm-like structure started at the level of the lower pharynx and later expanded down the body column, away from the oral pole. It was dependent on feeding and initially grew at a faster pace during the first 2 months, slowing down in later stages. Since the growth of the neoplasm-like structure impacts the mouth region, the decrease in rate of tissue expansion could be an indirect result of a lower food intake. The neoplasm-like structure will continued to grow and eventually engulfed the whole head of the animal. The animals could survive for some time in such a terminal state but eventually died due to their inability to feed (Fig. 6A-F).

**Fig. 7.**
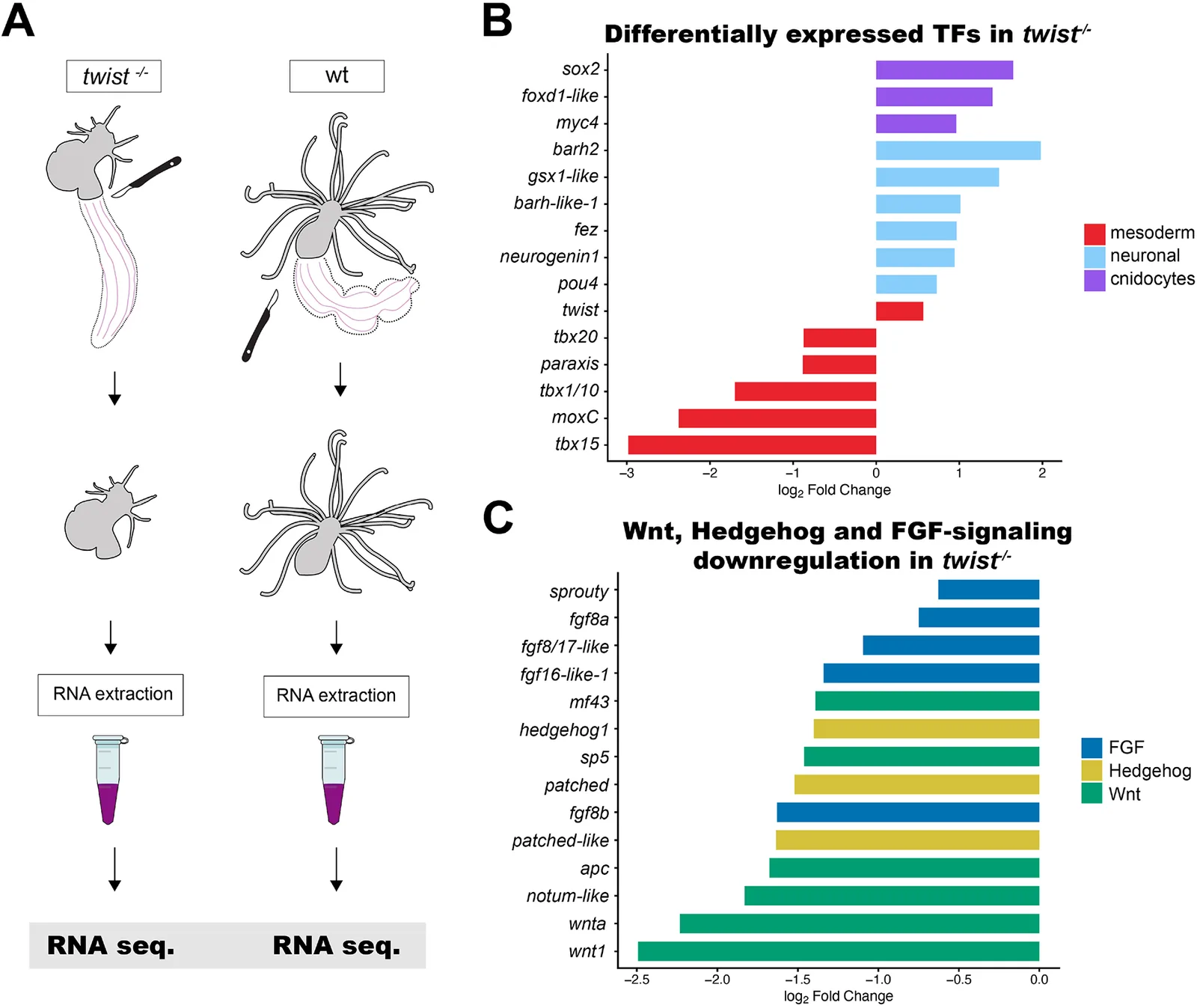
**Transcriptional changes in *twist*^−/−^ mutants.** (A) Schematic overview of bulk RNA-seq experimental design. Head tissue (including tentacles) from wild-type (WT) and *twist*^−/−^ animals exhibiting neoplasm-like development was collected for RNA extraction and sequencing. (B) Differentially expressed transcription factors in *twist*^−/−^ mutants relative to WT. Mesoderm-associated transcription factors are predominantly downregulated, while neuronal and cnidocyte-associated transcription factors show increased expression. (C) Downregulation of key developmental signalling pathways in *twist*^−/−^ mutants. Components of the Wnt, Hedgehog and FGF signalling pathways show reduced expression in mutant animals. Values represent log_2_ fold change relative to WT. Full lists of differentially expressed genes and transcription factors are provided in Tables S1 and S2. *n*=3 biological replicates per condition. Differential expression analysis performed using edgeR with TMM normalisation and quasi-likelihood F-test. Significance thresholds and fold-change cutoffs are provided in the Materials and Methods. Raw data deposited in GEO under accession number GSE329021.

To further characterise the downstream effects of Twist loss at the transcriptional level at the juvenile polyp stage, we analysed differential gene expression between wild-type and *twist* mutant animals exhibiting neoplasm-like development using head tissue (including tentacles) from animals exhibiting neoplasm-like development (three biological replicates per condition) (Fig. 7A; full list of differentially expressed genes in Table S1). We confirmed the observed downregulation of co-expressed mesodermal transcription factors such as *paraxis* and *tbx15* at the planula stage (Fig. 2C) also in the polyp stage (Fig. 7B). Moreover, we identified a broader downregulation of mesoderm-associated transcription factors, including *tbx1/10*, *tbx20* and *moxC* (Fig. 7B). We also observed a slight increase in *twist* expression in the mutant polyp. Genes associated with major developmental signalling pathways were significantly affected: multiple components of the Wnt signalling pathway, including *wnt1*, *wnta*, *sp5*, *notum-like*, *apc* and *rnf43*, associated with axial patterning, were strongly downregulated in *twist* mutants (Fig. 7C). Similarly, Hedgehog pathway components such as *hedgehog1*, *patched* and *patched-like* were also reduced. Notably, several FGF ligands, including *fgf8a*, *fgf8b*, *fgf8/17-like*, and *fgf16-like-1*, showed decreased expression, along with the feedback inhibitor *sprouty* (Fig. 7C).

Interestingly, we did not observe a strong or canonical proliferation gene signature or upregulation of oncogenes. Instead, several upregulated genes of extracellular matrix (ECM) molecules (e.g. laminin, collagen) or digestive enzymes (e.g. trypsin, peptidase) were consistent with the increased secretory/gland-like activity and/or ECM remodelling in the mutant tissue (Table S1). Moreover, neuronal and cnidocyte-associated transcription factors were predominantly upregulated, including genes such as *barh2*, *gsx1-like*, *pou4* and *sox2* (Fig. 7B; full list of differentially expressed transcription factors in Table S2). Together, this suggests an increase in gastrodermal and tentacle-specific cell types in the mutant neoplasm-forming tissue.

We next wished to know whether the two phenotypes – impaired secondary tentacle formation and epithelial neoplasm-like – are reproducible even after decapitation and regeneration. If the neoplasm-like is removed by bisection of the whole head, animals will regenerate a head and pharynx (Fig. 6G-P). Both WT animals and *twist^−/−^* mutants initiated tentacle regeneration simultaneously, yet, while WT animals regenerated all 16 tentacles at once, *twist^−/−^* mutants initially regenerated only 4-8 tentacles, reproducing the initial phenotype (Fig. 6H,I,M,N). Later, the some mutants formed an additional 2-4 tentacles, which were often malformed or crippled (Figs S3B and S6). Thus, the programme to generate tentacles in every other mesenterial segment is not affected in the mutant, but the formation of additional tentacles and the subdivision in pseudo-segments is also impaired during regeneration. Moreover, epithelial neoplasm-like outgrowths reappeared, often after several weeks, although onset was variable and occurred at different time points (Fig. 6P). These observations strongly suggest that the defects are reproducible and robust and, hence, likely to be of genetic cause.

**Fig. 8.**
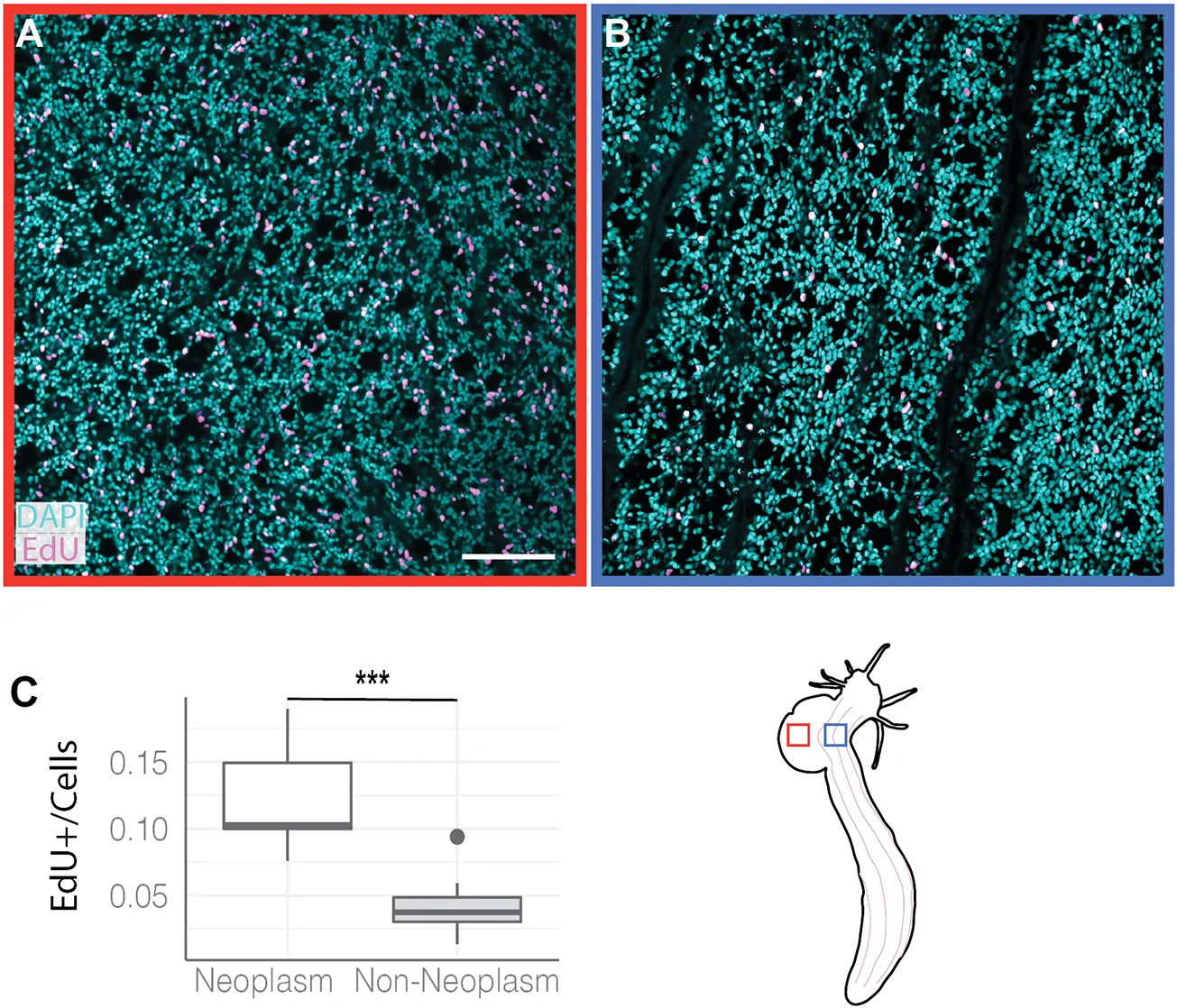
**EdU staining shows increased proliferation of neoplasm-like tissue in *twist^−/−^* animals.** (A) EdU (magenta) and DAPI (cyan) staining of the neoplasm-like side, showing actively dividing cells in the expanding region. (B) EdU and DAPI staining of the non-neoplasm-like side, showing less cell division. (C) Quantification of the ratio of EdU-positive cells to total cell nuclei, indicating a higher cell division ratio on the bubble side compared to the non-bubble side. The accompanying sketch illustrates the sample locations: red for neoplasm-like and blue for non-neoplasm-like. *n*=16 *twist*^−/−^ animals, each serving as its own control (neoplasm-like side versus unaffected side). ***P<0.001 (two-tailed Welch's *t*-test). Box plots show median values (middle bars) and first to third interquartile ranges (boxes); whiskers indicate 1.5× the interquartile ranges; dot indicates outlier. Scale bar: 50 μm.

**Fig. 9.**
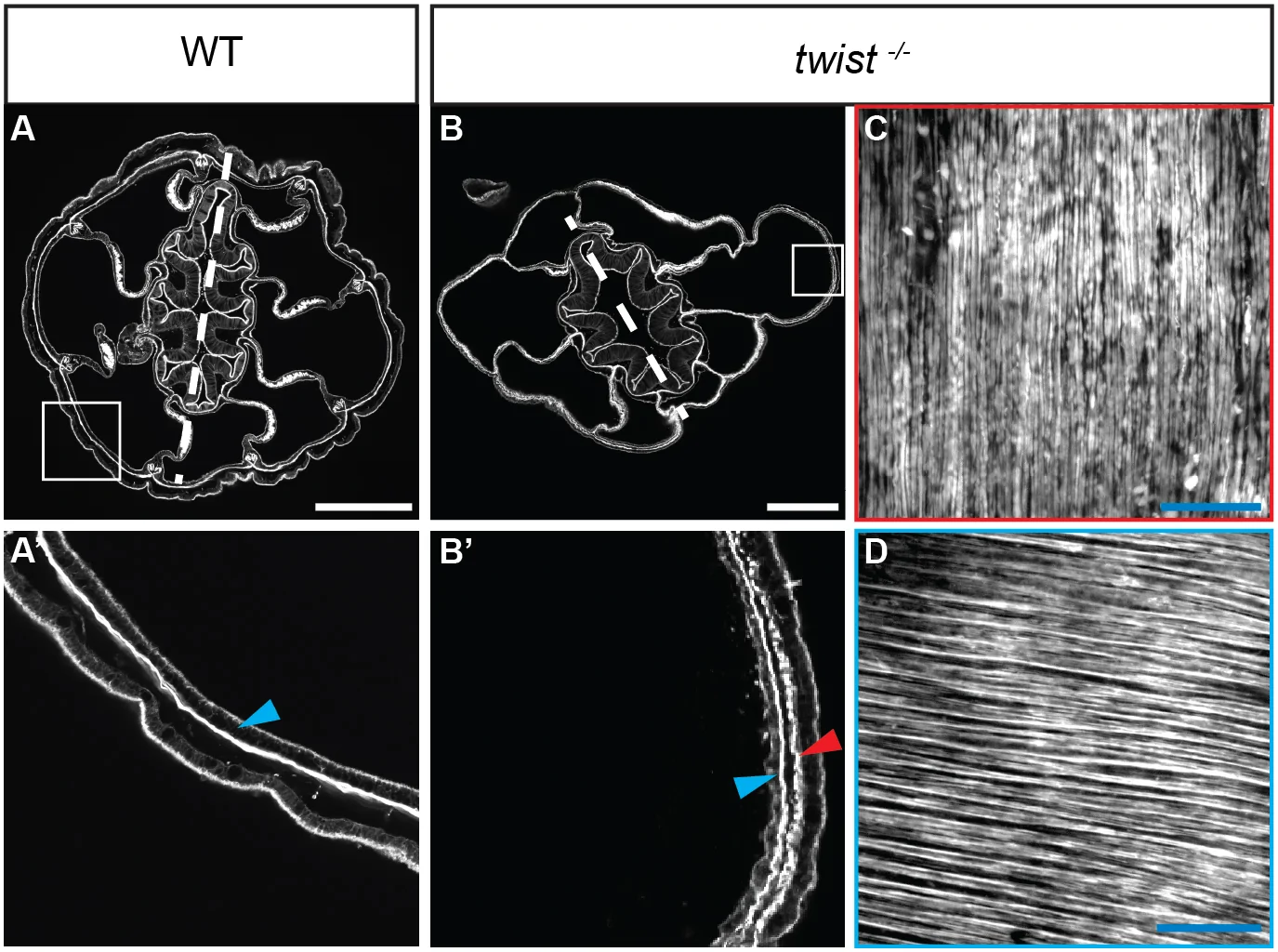
**Sagittal sections of the neoplasm-like reveal ectopic longitudinal ectodermal muscle cells in *twist^−/−^* animals.** (A) Phalloidin staining of a sagittal cross-section of a wild-type (WT) adult at the height of the pharynx, showing the anatomical features of the region. (A′) Close-up view of the inset shown in the boxed area in A, with a blue arrowhead indicating the mesodermal muscle layer. (B) Phalloidin staining of a sagittal cross-section of an adult twist mutant at the height of the pharynx, highlighting the morphological differences compared to WT. (B′) Close-up view of the inlet in the boxed area in B, with a blue arrowhead indicating the mesodermal muscle layer and a red arrowhead indicating the ectodermal muscle layer. (C) Horizontal close-up view of the ectodermal muscle layer of the neoplasm-like, revealing its longitudinal alignment. (D) Horizontal close-up view of the mesodermal muscle layer of the neoplasm-like, showing its horizontal alignment. Dashed lines are used to indicate the directive axis. The ectopic ectodermal muscle phenotype was observed in multiple *twist*^−/−^ animals across the course of the study. Panels show representative individuals. Scale bars: 500 μm (A,B); 50 μm (C,D).

To test whether the neoplasm-like is due to excessive proliferation or to a balloon-like inflation of the epithelial tissue, we conducted an EdU labelling experiment and compared the side of the animal with the neoplasm-like versus the normal side within the same mutant polyp. We examined 16 *twist*^−/−^ animals and found that the neoplasm-like side had a higher proliferation ratio than the unaffected side within the same animal (Fig. 8). Sagittal sections of the neoplasm-like area revealed that the mesoglea, which separates the gastrodermis and ectoderm, became considerably thicker during body wall expansion. Notably, in addition to the gastrodermal circular muscle, the sections also revealed an ectopic ectodermal muscle. This is remarkable, as usually only tentacles have ectodermal muscles (Fig. 9A,B) (i.e. the longitudinal retractor muscles) (Jahnel et al., 2014). When investigated in more detail, we found that the ectopic muscle layer was longitudinally aligned, mirroring the orientation of the ectodermal muscle layer in the tentacles (Fig. 9C,D).

**Fig. 10.**
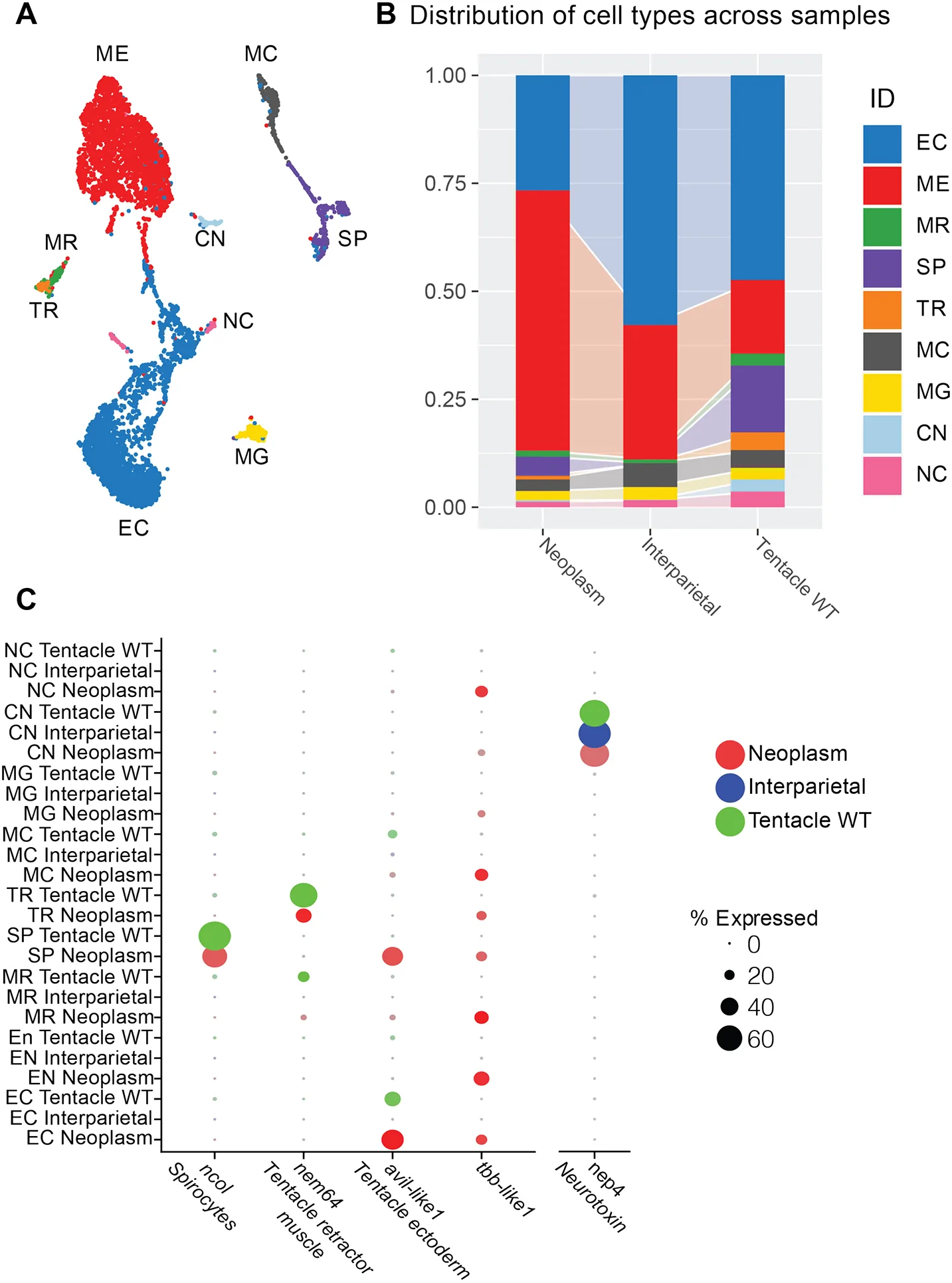
**scRNA-seq analysis of neoplasm-like reveals a cellular profile reminiscent of tentacles.** (A) UMAP projection of integrated single-cell transcriptomes from neoplasm-like, tentacle and interparietal body wall tissues. Cell clusters were annotated based on known marker expression. (B) Cell type composition of each tissue, showing that spirocyte and tentacle retractor clusters are absent in the interparietal sample but present in both the neoplasm-like and tentacle. (C) Dot plot showing expression of tentacle-specific genes (*ncol*, *nem64*, *avil-like1*) in neoplasm-like and tentacle samples. *tbb-like1* is specifically upregulated in the neoplasm-like, while *nep4* (a general cnidocyte marker) is expressed across all three tissues. Each library was generated from a single individual. *twist*^−/−^ tentacle and neoplasm-like libraries were generated in this study; WT tentacle and interparietal libraries were obtained from Steger et al. (2022). Sequencing data deposited in GEO under accession number GSE308593. CN, cnidocytes; EC, ectoderm; MC, mature cnidocytes; ME, mesoderm; MG, mucus glands; MR, mesentery retractor muscle; NC, not categorised; SP, spirocytes; TR, tentacle retractor muscle.

This suggested that the neoplasm-like is not only an epithelial overgrowth but may have adopted a tentacle-like identity, in line with the bulk RNA-seq analyses (Fig. 7). To further test this, we generated single cell (sc) RNA-seq libraries from dissected neoplasm-like tissue and compared them to WT tentacle and body wall samples. We then clustered the combined dataset and annotated major cell types based on known markers (Fig. 10A). While the UMAP projection does not distinguish cells by sample origin, analysis of the cell type distribution revealed that spirocytes and tentacle retractor muscle cell types were absent from the interparietal tissue but present in both the neoplasm-like and tentacle samples (Fig. 10B). Consistently, tentacle-specific transcripts such as *nem64* (a transcription factor associated with the retractor muscle; Cole et al., 2023), *avil-like1* (tentacle-specific ectoderm marker) and *ncol* (a spirocyte specific minicollagen) were expressed exclusively in the neoplasm-like and in tentacle samples, but not in the WT-interparietal control sample (Fig. 10C). We also observed an upregulation of *tbb-like1* specifically in the neoplasm-like-interparietal tissue (Fig. 10C) While this β-Tubulin isoform was broadly expressed across nearly all clusters, it showed enrichment in a subset of neural subtypes (Fig. S6). Together with α-Tubulin, β-Tubulin forms the essential component of microtubules. Although the role of *tbb-like1* in the neoplasm-like is unclear, its upregulation may be related to the higher rate of cell division and the transformation into a neoplasm-like outgrowth (see Discussion). These data suggest that, in juvenile *twist* mutants a neoplasm-like structure emerges by epithelial hyperproliferation and adopts a tentacle-like transcriptional profile.

## DISCUSSION

### *twist* in *N. vectensis* is not essential for initial germ layer specification and mesentery segments

The Twist family of bHLH transcription factors was originally identified in *Drosophila melanogaster* as a central regulator of mesoderm specification and muscle development (Thisse et al., 1987, 1988; Leptin and Grunewald, 1990). Twist homologues are present throughout metazoans, from cnidarians to vertebrates (Yasui et al., 1998; Corsi et al., 2000; Spring et al., 2000; O'Rourke and Tam, 2002; Martindale et al., 2004; Wu et al., 2008), and share highly conserved DNA-binding and dimerisation domains (Ellenberger et al., 1994; Wu et al., 2008). Despite this structural conservation, functional roles of Twist vary considerably across lineages (Davis et al., 1990; Ma et al., 1994), showing its regulatory capacity and context-dependent ability to form hetero- and homodimers, as Twist functions as an obligate dimer that cannot bind DNA as a monomer (Castanon et al., 2001; Maia et al., 2012; Chang et al., 2015; Bouard et al., 2018). In *N. vectensis*, *twist* expression initiates at the post gastrulation stage in the mesodermal compartments surrounding the developing pharynx that later give rise to the mesenteries (Martindale et al., 2004). In contrast to *Drosophila*, loss of *twist* does not impair gastrulation or germ layer specification, nor does it affect formation of the first eight mesentery segments, which are regulated by BMP signalling and downstream Hox genes (Saina et al., 2009; Genikhovich et al., 2015; He et al., 2018). This observation aligns with several bilaterian systems in which *twist* acts after mesoderm specification rather than as an initial determinant factor (Molkentin et al., 1995; Harfe et al., 1998; Yasui et al., 1998; Spring et al., 2000), suggesting that the strict requirement for Twist during early mesoderm formation in *Drosophila* may represent a derived condition. Although gastrodermal cells in *Nematostella* undergo partial epithelial-mesenchymal transition (EMT) during gastrulation (Kraus and Technau, 2006; Pukhlyakova et al., 2019), our results do not support an ancestral role of *twist* in this process.

### Twist regulation by Wnt, Notch and BMP signalling in *N. vectensis*

**Fig. 11.**
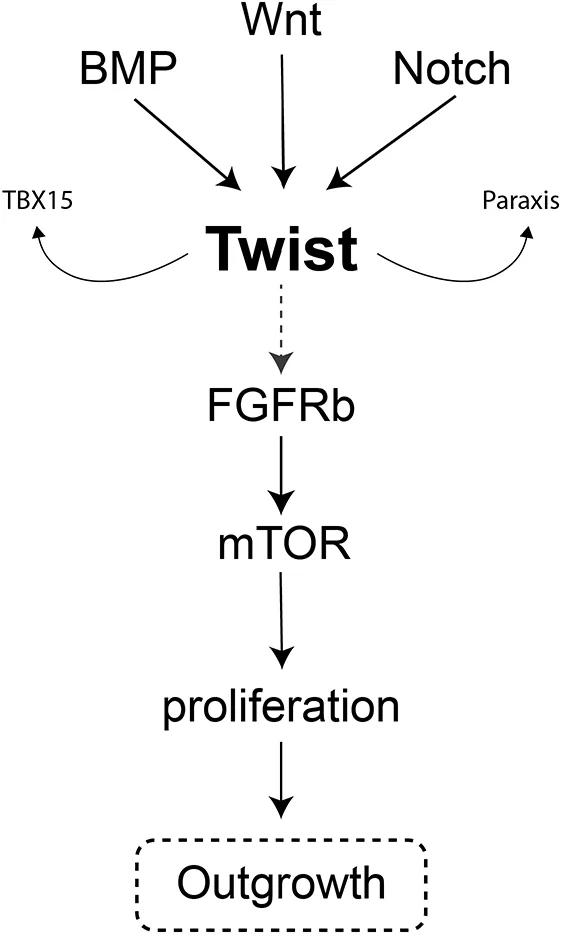
**Proposed regulatory model of Twist function during tentacle morphogenesis in *N. vectensis*.** *twist* expression is regulated by Wnt, BMP and Notch signalling. Twist acts upstream of mesoderm-associated transcription factors, including *paraxis* and *tbx15*. During post-embryonic development, Twist maintains FGFRb expression and supports activation of TOR signalling, promoting localised proliferative outgrowth. The dashed arrow indicates an indirect or unresolved regulatory interaction.

Our results place *twist* within a regulatory network integrating several developmental signalling pathways. Wnt signalling is both necessary and sufficient for *twist* activation, consistent with regulatory interactions described in vertebrate systems (Howe et al., 2003; Reinhold et al., 2006; Goodnough et al., 2016; Ji et al., 2019). In addition, inhibition of Notch or BMP signalling results in strong reduction of *twist* expression (Fig. 11). In bilaterians, Notch has been shown to modulate or directly regulate *twist* depending on context (Tapanes-Castillo and Baylies, 2004; Tian et al., 2015; Wang et al., 2020), while BMP signalling has also been reported to regulate Twist expression in vertebrate mesoderm in a stage dependent manner (Hornik et al., 2004). Our findings place *twist* downstream of Wnt, Notch and BMP signalling in *Nematostella*, pathways that govern mesenterial and axial patterning (Genikhovich et al., 2015; Saina et al., 2009). In contrast, inhibition of MAPK or Hedgehog signalling had no detectable effect on *twist* expression. Although MAPK-dependent post-translational regulation of Twist has been described in vertebrates (Hong et al., 2011; Li et al., 2013), our results suggest that MAPK and Hedgehog are not major transcriptional regulators of *twist* in *Nematostella*.

Our results further show that *paraxis* and *tbx15*, both co-expressed with *twist*, are strongly downregulated in the *twist^−/−^* mutants, indicating that *twist* functions upstream and is required for their normal expression (Fig. 11). *Paraxis* belongs to the Twist-related family of bHLH transcription factors and is frequently co-expressed with *twist* in bilaterian systems (Barnes and Firulli, 2009; Wilson-Rawls et al., 2004), with evidence from brachiopods suggesting that *paraxis* expression can follow *twist* activation (Passamaneck et al., 2015). In contrast, the relationship between Twist and T-box genes does not appear to follow a consistent pattern across lineages, suggesting that the gene regulatory network architecture is not conserved in a uniform manner (Isaac et al., 2000; Rose et al., 2022).

### Transcriptional and signalling changes in twist mutants

The coordinated downregulation of mesoderm-associated transcription factors including *paraxis*, *tbx15*, *tbx1/10*, *tbx20* and *moxC*, alongside the upregulation of neuronal and cnidocyte-associated transcription factors suggests a shift in tissue composition or identity rather than a simple block in differentiation. Given that Twist is expressed post-gastrulation, this is unlikely to reflect a role in initial germ layer specification, but rather a function in maintaining mesodermal identity in differentiated tissues, consistent with described roles for Twist in mesodermal maintenance across bilaterians (Baylies and Bate, 1996; Castanon and Baylies, 2002).

The concurrent downregulation of components from multiple signalling pathways including Wnt ligands and pathway regulators, Hedgehog components, and several FGF ligands along with the feedback inhibitor Sprouty points to a broader reorganisation of the signalling environment. Given that these pathways play central roles in tissue patterning and morphogenesis, their reduction more likely reflects the loss or disorganisation of signalling competent mesodermal tissue rather than direct transcriptional control by Twist. In particular, FGF signalling has been implicated in coordinating morphogenetic processes in *Nematostella* and across metazoans (Matus et al., 2007), and its reduction is consistent with the impaired tentacle outgrowth observed in *twist* mutants. Similarly, the downregulation of Wnt pathway components is notable given the established role of Wnt signalling in axial patterning and post-gastrulation morphogenesis in *Nematostella* (Kusserow et al., 2005).

Together, these results support a model in which Twist stabilises mesodermal identity in differentiated tissues, and where its loss leads to both a compositional shift toward neural and cnidocyte fates reflected by the upregulation of transcription factors including *sox2*, *pou4*, *barh2* and *gsx1-like* (Marlow et al., 2012; Richards and Rentzsch, 2015; Steger et al., 2022) and a disruption of the signalling landscape required for coordinated growth and patterning.

### Twist controls the morphogenesis of secondary tentacles

A key finding of this study is that *twist* is required for the timely emergence and morphogenesis of secondary tentacles during post embryonic development. Primary tentacles form largely normally, consistent with previous work describing distinct phases of tentacle addition (Fritz et al., 2013; Ikmi et al., 2020), whereas *twist* mutants exhibit delayed secondary tentacle addition, reduced tentacle length and altered oral disc morphology. At the molecular level, we observe delayed activation of TOR signalling, as indicated by reduced pRPS6 localisation, and weakening of *fgfrb* expression during secondary tentacle initiation. FGFRb has previously been shown to mediate localised TOR activation during tentacle outgrowth (Ikmi et al., 2020). The spatial separation between mesodermal *twist* expression and ectodermal TOR activation supports an indirect model in which Twist promotes localised tentacle outgrowth through FGF-dependent TOR signalling.

This positioning of *twist* upstream of FGF-dependent TOR activation suggests that its function may be part of a conserved growth regulatory signalling network. Notably, *twist* mutants fail to form microcnemes, which are incomplete mesentery folds that demarcate prospective tentacle positions within segments (Ikmi et al., 2020). Proper mesenterial segmentation is crucial for tentacle positioning (He et al., 2018). The failure to form microcnemes likely disrupts spatial organisation, contributing to reduced and mispatterned secondary tentacles. Although the precise causal relationship between microcneme loss and altered TOR/FGF dynamics remains unresolved, our data suggest that Twist coordinates morphogenetic competence and localised growth activation rather than broadly controlling cell differentiation.

### Twist does not regulate muscle specification in *Nematostella*

Despite morphological abnormalities in mutant tentacles, muscle layers form with appropriate orientation and organisation. This contrasts with roles described for Twist in certain bilaterian systems, including nematodes and vertebrates, where it regulates myogenic differentiation or prevents premature muscle specification (Bialek et al., 2004; Corsi et al., 2000). In *Nematostella*, recent work has demonstrated that the bHLH transcription factor Nem64 is specifically required for the specification of the ectodermal tentacle retractor muscle lineage, while other muscle populations remain unaffected (Cole et al., 2023). This indicates that *twist* is not a general regulator of muscle specification, but instead that distinct bHLH factors control individual muscle subtypes.

### Evolutionary implications

The positioning of *twist* downstream of Wnt/BMP/Notch signalling and upstream of FGF/TOR links axial patterning inputs to localised growth activation during secondary tentacle formation. In bilaterians, FGF signalling from organising centres such as the AER promote appendage outgrowth, with Twist participating in appendage-associated mesenchymal programmes (O'Rourke and Tam, 2002; Zúñiga et al., 2002; McQueen and Towers, 2020). Our findings place *twist* in a comparable regulatory context in *Nematostella*, linking mesodermal transcriptional control to FGF-dependent growth activation. A schematic summarises the regulatory architecture in *Nematostella*, positioning Twist downstream of Wnt, BMP and Notch signalling and linking mesodermal transcriptional programmes to FGF–mTOR-dependent outgrowth (Fig. 11). Although tentacles and vertebrate limbs are not homologous structures, the conserved positioning of Twist upstream of FGF-dependent growth suggests that this interaction represents an evolutionarily ancient mechanism controlling appendage morphogenesis.

### *twist* mutants develop oral body wall neoplasm-like structures with a tentacle-like profile

Older juvenile twist mutants develop proliferative epithelial outgrowths at the oral pole, between mesenteries at the level of the pharynx where *twist* is normally expressed (Fig. 6). scRNA-seq revealed that these neoplasm-like structures adopt a tentacle-like transcriptional profile, including the presence of spirocytes and expression of the tentacle retractor muscle marker *nem64*. Consistent with this, longitudinal musculature characteristic of the ectodermal tentacle lineage is also present. The emergence of proliferative tissue with tentacle-like molecular identity but aberrant morphology suggests that Twist is required not for specification of tentacle cell types per se, but for spatially restricting growth and coordinating proper morphogenesis.

These findings indicate a partial uncoupling between tissue identity and morphogenesis, in which appropriate cell types are specified but fail to organise into functional appendages. The absence of microcnemes in *twist* mutants may contribute to this phenotype by disrupting spatial boundaries that normally constrain localised growth. Supporting this interpretation, differential expression analysis identified upregulation of proliferation-associated genes, including the β-tubulin isoform *tbb-like1*, which is not expressed in WT tentacles or body wall tissue. In addition, comparison with previously described cnidarian tumours revealed shared upregulation of the cancer-associated factor *TCTP-like1* (Domazet-Lošo et al., 2014; Table S3). Similar neoplasm-like outgrowths have also been observed in *Nematostella* following disruption of BMP signalling (Knabl et al., 2025), consistent with our finding that *twist* expression depends on BMP signalling. This supports a model in which disruption of upstream patterning inputs can lead to loss of spatial growth control. While these neoplasm-like structures do not exhibit features of malignant transformation, their transcriptional profile supports the interpretation that Twist is required to spatially restrict proliferative growth and maintain epithelial tissue organisation. Together, these findings identify Twist as a key regulator of spatial growth control, ensuring that appendage formation occurs at defined positions rather than as unpatterned proliferative outgrowth.

## MATERIALS AND METHODS

### Animal husbandry

The *Nematostella* polyps were maintained under the conditions outlined in previous studies (Fritzenwanker and Technau, 2002; Genikhovich and Technau, 2009b) at a temperature of 18°C and predominantly in the absence of light. The induction of spawning was facilitated through the combined influence of light and increased temperature (Fritzenwanker and Technau, 2002) – the embryos being incubated at a temperature of 21°C. Animals were of mixed sex; sex was not explicitly controlled in experimental groups.

### CRISPR/Cas9 mutagenesis

CRISPR/Cas9-induced deletion was carried out as previously described (Ikmi et al., 2014). Using CRISPOR, (Concordet and Haeussler, 2018) a guide RNA (gRNA) targeting the HLH domain of *Nematostella twist* (**CCC**ACGTTACCTTCCGATAAACT; PAM motif in bold) was designed, synthesised with the MEGAshortscript T7 kit, and purified with 3 M sodium acetate/ethanol. Then 500 ng/μl of gRNA was co-injected into *Nematostella* embryos along with 1 μg/μl recombinant Cas9 protein (PacBio) and raised at 21°C. To determine the efficiency of the CRISPR/Cas9-induced deletion, tentacle tips from injected primary polyps were taken and genomic DNA extracted (gDNA). Each tentacle tip was treated in 20 μl DNA extraction buffer (10 mM Tris-HCl pH 8, 50 mM KCl, 0.3% Tween 20, 0.3% NP40, 1 mM EDTA) with 1 μg/μl proteinase K (Thermo Fisher Scientific) at 58°C, and inactivated at 98°C for 20 min. A 2 μl gDNA sample was used for melting curve PCR analysis. After the efficiency of the gRNA was confirmed, F0 injected animals were raised to sexual maturity and crossed with WT animals. F1 progeny with detected indels were sequenced and pooled by mutation type, and interbred at sexual maturity to yield F2 homozygous mutants.

### Whole mount *in situ* hybridisation (WMISH)

WMISH carried out as previously described (Genikhovich and Technau, 2009b), but with minor adjustments to improve the detection of the *twist* gene. Embryos were fixed in 4% paraformaldehyde (PFA) in phosphate-buffered saline (PBS) for 1 h at room temperature (RT), then washed five times with 0.2% Tween in PBS at RT. The embryos were then washed once with 50% methanol in PBS containing 0.2% Tween before being transferred to 100% methanol and kept at −20°C until further processing. Hybridisation with 1 ng riboprobe/μl was carried out overnight at 63°C, followed by detection with an Alkaline Phosphatase-conjugated anti-digoxigenin antibody (anti-digoxigenin-AP, Fab fragments, Roche, 11093274910, 1:2000). Upon staining, the embryos underwent a series of five washes in PBS/0.2% Tween (PTw). Subsequently, the embryos were immersed in PBS containing 2% w/v Triton X-100 until the background reached an acceptable threshold. The process was repeated until a robust signal was detected.

### Drug treatments

Embryos were incubated for 24 h in *Nematostella* medium containing the respective drug dissolved in DMSO. Treatments were predominantly carried out at 48-72 hpf, except for one batch treated with Azakenpaullone (AZ) at 24-48 hpf to assess potential early effects of Wnt signalling on *twist* expression. The following concentrations were used: ICRT14 (Wnt/β-catenin inhibitor, 50 μM; Sigma-Aldrich), Cyclopamine (Smoothened inhibitor, 10 μM; Sigma-Aldrich), U0126 (MEK inhibitor, 20 μM; Sigma-Aldrich), K02288 (BMP receptor inhibitor, 6 μM; Sigma-Aldrich), LY-411575 (Notch signalling inhibitor, 50 μM; MedChemExpress, HY-50752) and AZ (GSK3β inhibitor, 5 μM; Sigma-Aldrich). Control embryos were incubated in equivalent concentrations of DMSO under identical conditions. After incubation, embryos were fixed and processed for *in situ* hybridisation analysis as described above.

### Differential gene expression analysis

Total RNA was extracted from head tissue (including tentacles) of WT and *twist* mutant animals exhibiting neoplasm-like development using TRIzol reagent (Invitrogen), with three biological replicates per condition. Before extraction, tissues were flash-frozen in liquid nitrogen and mechanically homogenised using a mortar and pestle in the presence of TRIzol reagent to ensure efficient disruption and preservation of RNA integrity. Raw sequencing reads were trimmed using Trimmomatic v0.39, applying a sliding window approach (4 bp window, minimum average quality score of 15) and retaining reads with a minimum length of 75 bp. Trimmed reads were aligned to the *N. vectensis* reference genome (Zimmermann et al., 2023) using STAR v2.7.3a with default parameters. Gene-level read counts were obtained using featureCounts from the Subread package v2.0.0, counting paired-end reads with a minimum mapping quality of 20. Differential gene expression analysis was performed in R using the edgeR package, applying TMM normalisation and a quasi-likelihood F-test to assess differences between conditions.

### Immunohistochemistry and staining

For EdU labelling, the Click-iTTM EdU Cell Proliferation kit (Invitrogen) was used. Primary polyps at the 5+6 tentacle bud stage were incubated for 30 min at RT with 50 µM EdU in 16% artificial sea water (ASW). The ‘bubble’ and unaffected adult tissues, on the other hand, were treated for 1 h at RT. All specimens were then anesthetised with 7% MgCl_2_ in 16% ASW. The primary polyps were fixed in 4% PFA in PBS for 1 h at RT, while adult tissues were fixed overnight at 4°C. The tissues were then premetallised, blocked, and the Click-it reaction was performed following standard protocols. In cases where additional antibody staining was required, another round of permeabilisation and blocking was carried out before an overnight incubation at 4°C with rabbit-anti-pRPS6 (Cell Signaling Technology, 4858; 1:1000). Anti-NvNcol-3 antibody was kindly provided by the Holstein laboratory (University of Heidelberg, Germany). The tissues were then washed and counterstained overnight at 4°C with anti-rabbit secondary antibodies (Alexa-Fluor 488, Invitrogen, A32731; 1:2000) and 14 µM DAPI in a buffer of PBS supplemented with 10% goat serum, 1% DMSO and 0.1% Triton X-100. For Phalloidin staining, polyps and adult tissues were fixed according to their developmental stage, then stained with 2.2 µM Alexa Fluor^®^ 488 Phalloidin (Invitrogen, A12379) for 2 h at RT followed by five washes in PBT (0.5% Triton X-100 in PBS). Adult polyps were embedded in 10% gelatin in PBT and sectioned at 70-100 μm thickness using a Leica VT1200 vibrating blade microtome.

### Cnidocyte staining

Primary polyps were anaesthetised with 7% MgCl_2_/16 ASW for 10-20 min, as per the protocol modified from Wolenski et al. (2013). 4% formaldehyde (FA)/16% ASW/0.5 M EDTA was used for a quick pre-fixation, and the same solution was used for a 1 h fix at 4°C after that. Samples were rinsed with Tris-EDTA washing buffer (10 mM Tris, 10 mM EDTA, 10 mM NaCl, pH 7.6) at least three times after fixation. After that, they were incubated with 14 µM DAPI/0.66 µM Alexa Fluor^®^ 633 Phalloidin (Invitrogen, A22287) in the washing buffer overnight at 4°C. Before mounting, a series of five Tris-EDTA buffer washes were performed after incubation. All samples were finally incubated overnight at 4°C in Vectashield^®^ antifade mounting solution for maximum preservation and image quality.

### scRNA-seq

Adult *Nematostella* tissues were prepared for scRNA-seq, including tentacles, interparietal and bubble (neoplasm-like). The tissues were treated with TrypLE™ Select (Thermo Fisher Scientific, A1217701) for 4 h to assist dissociation into a single-cell solution. The samples were gently pipetted up and down every 30 min to aid in dissociation, with extra care taken to minimise suctioning, especially during the first hour of the incubation. This procedure was repeated until a homogeneous single-cell suspension was obtained. Cell viability and counts were then determined using a Nexcelom Cellometer X2, and suspensions were diluted to 1000-2000 cells/μl. To ensure cell viability, the samples were stored on ice until they were ready for processing. The single-cell suspensions were put into a 10x Genomics single-cell platform, and libraries were produced using Chromium Next GEM Single Cell 3′ Kit v3.1 (dual indexed) reagents according to the manufacturer's instructions, with the goal of recovering 7000 cells per library. The reads were mapped and aligned to the most recent *Nematostella* genome (Zimmermann et al., 2023) using the CellRanger 5.1.0 pipeline with the default settings.

### Single-cell analysis

Single-cell transcriptomic analysis was performed using the Seurat R package v5.2.1 (Butler et al., 2018; Stuart et al., 2019). Raw UMI count matrices from libraries (‘tentacle twist’, ‘interparietal’, ‘neoplasm-like’, ‘tentacle WT’) were processed as follows. Quality control filters retained cells with 200-2500 detected genes and fewer than 10,000 transcripts per cell. Highly variable genes were identified using the ‘vst’ method, and data were normalised using ‘LogNormalize’. Gene IDs were updated to the *N. vectensis* gene model annotations using a custom mapping table. To integrate datasets (‘Tentacle twist’, ‘Interparietal’, ‘Neoplasm-like’, ‘tentacle WT’), ‘FindIntegrationAnchors’ and ‘IntegrateData’ were used followed by scaling principal component analysis (PCA) (50 components). Uniform manifold approximation and projection (UMAP) was used for dimensionality reduction. Clustering was tested over resolutions ranging from 0.1 to 0.8 with final analyses performed at 0.8, using shared nearest neighbour graph. UMAP ‘DimPlots’ were used for library comparisons, ‘DotPlots’ were used to illustrate expression patterns and cluster identities were assigned based on marker gene expression. Differential gene expression between libraries was assessed using Seurat's ‘FindMarkers’ function (log2 fold-change>0.25, detection rate>25%). Relative cell type proportions were calculated independently for each library. One cluster (referred to as LA cluster), identified by its strong enrichment in *twist* mutant tentacles and mixed marker expression consistent with a dissociation artefact, was excluded from proportional analyses. Exclusion of this cluster resulted in only minor proportional changes and did not affect biological conclusions.

### AI declaration

AI tools (ChatGPT/Claude) were used to assist with language editing of the manuscript. The authors subsequently reviewed and edited the content as necessary and take full responsibility for the publication's final content. All original writing, data collection, and analysis were performed by the authors.

### Data availability

Raw and processed sequencing data supporting this study have been deposited in the NCBI Gene Expression Omnibus (GEO). scRNA-seq data are available under accession number GSE308593, with individual libraries available as GSM9248943 (Twist KO tentacle) and GSM9248944 (Twist KO neoplasm-like). Bulk RNA-seq data are available under accession number GSE329021.

For comparative analyses, we also integrated publicly available datasets from Steger et al. (2022) (GSE154105, GSE200198) and Cole et al. (2023) (GSE154477). A detailed overview of all datasets is provided in Table S4.

## Supplementary Material



10.1242/develop.205340_sup1 Supplementary information

Table S1. This table contains the full list of Differentially expressed genes derived from bulk RNAseq between twist-/- mutant neoplasm tissue versus wildtype body wall tissue. See also Fig. 7 for a selection of DEGs.

Table S2. This table contains the list of differentially expressed transcription factor genes from bulk RNAseq between twist-/- mutant neoplasm tissue versus wildtype body wall tissue. See also Fig. 7.

Table S3. This table contains the list of differentially expressed genes coding for tumor markers and oncogenes from scRNAseq between twist-/- mutant neoplasm tissue versus wildtype parietal body wall tissue and tentacle tissue. See also Fig. 7.

Table S4. This table contains an overview of all sequencing datasets used in this study with corresponding accession numbers.
