# Supplementary material for: Loss of Twist impairs tentacle development and induces epithelial neoplasia in the sea anemone Nematostella vectensis
Source: Development. 2026 Aug 20;153(17):dev205340. doi: 10.1242/dev.205340 (PMC13539428; doi:10.1242/dev.205340)
Supplement: Supplementary file 1 [file develop-153-205340-s1.pdf]

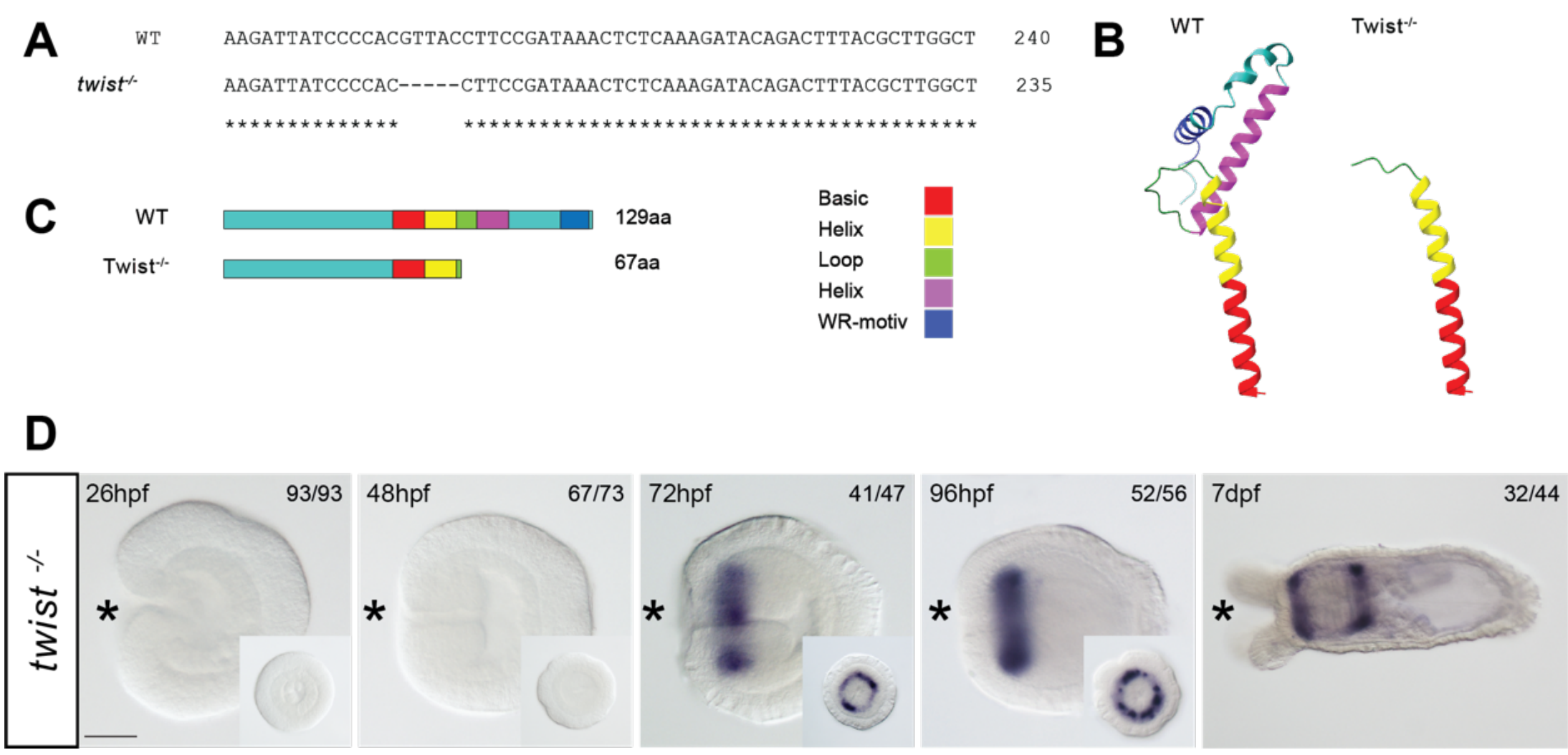

Fig. S1. Sequence and structural comparison of WT and Twist$^{-/-}$ proteins. (A) Segment alignment of WT and *twist*$^{-/-}$ nucleotide sequences showing a 5 bp deletion. **(B)** Alphafold predicted 3D structures of the bHLH domain of the WT and Twist$^{-/-}$ proteins (Jumper et al., 2021) **(C)** Schematic representation of the WT and Twist$^{-/-}$ proteins with colour-coded functional domains. WT protein is 129 amino acids long; Twist$^{-/-}$ protein is 67 amino acids long. **(D)** Whole Mount In Situ Hybridization (WMISH) of *twist* in the *twist*$^{-/-}$ mutant at different developmental stages. Asterisk indicates oral pole of the animals. Scale bar is 50µm. Hours post fertilization (hpf). Days post fertilization (dpf). n of total animals,values per timepoint: 26 hpf: 93; 48 hpf: 73; 72 hpf: 47; 96 hpf: 56; 7 dpf: 44. Experiment repeated multiple times with similar results; panel shows representative data from a single experiment.

Development • Supplementary information

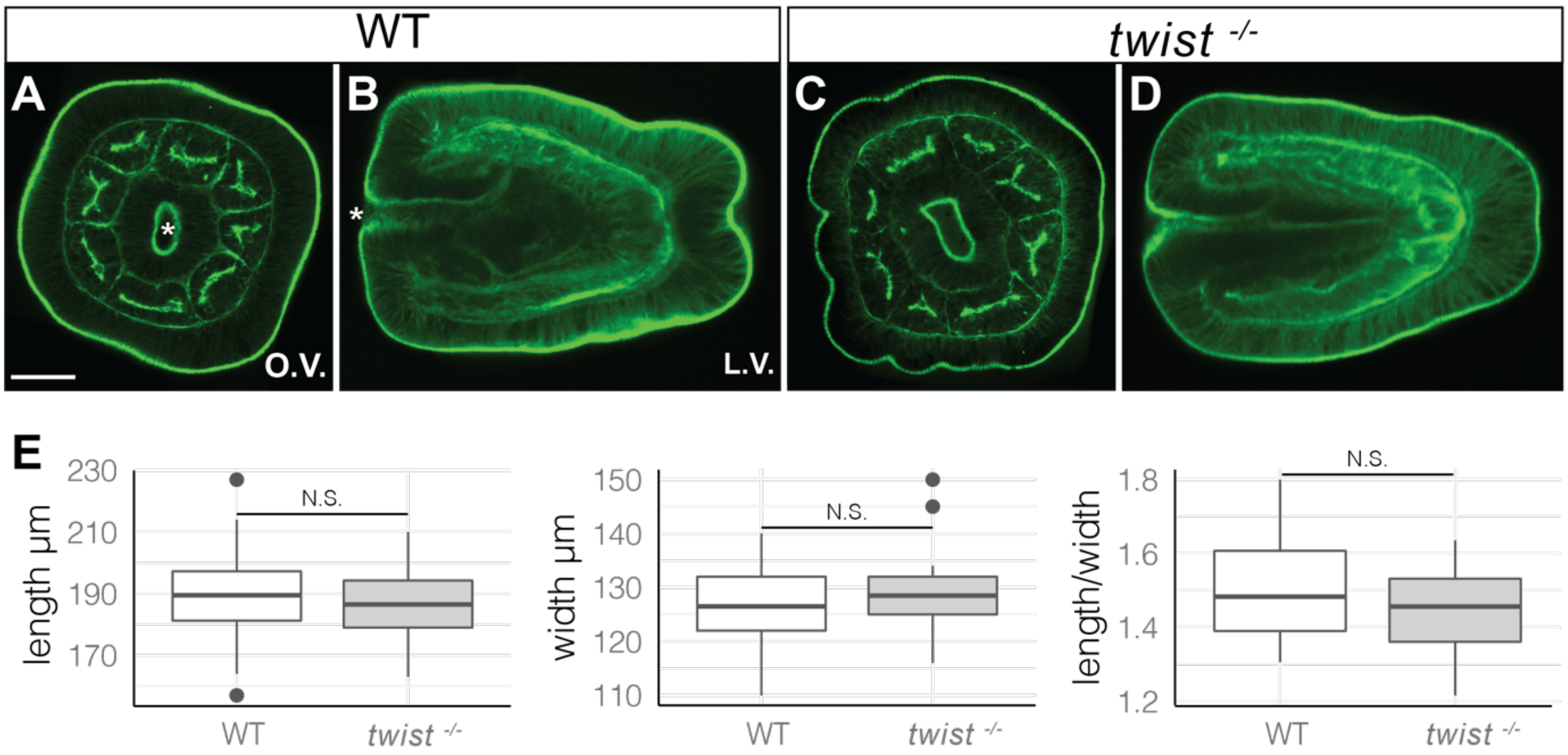

Fig. S2. Pre-mesenterial segment development and quantification of body proportions in WT and *twist*$^{-/-}$ animals.

(A, B) Oral and lateral confocal projections of a 4-day-old larva stained with phalloidin, showing the eight pre-mesenterial segments. **(C, D)** Oral and lateral views of a *twist*$^{-/-}$larvae at the same stage. Asterisks mark the oral pole. **(E)** Box plots quantifying length, width, and length-to-width ratio for WT and *twist*$^{-/-}$ larvae. Scalebar: 50 µm. n = 20 animals per genotype for panel E (body proportions). Panels A–D show single representative animals; 10 animals per genotype were examined with similar results. Single experiment. Box plots show median with interquartile range. Statistical comparisons by two-tailed Welch's t-test.

Development • Supplementary information

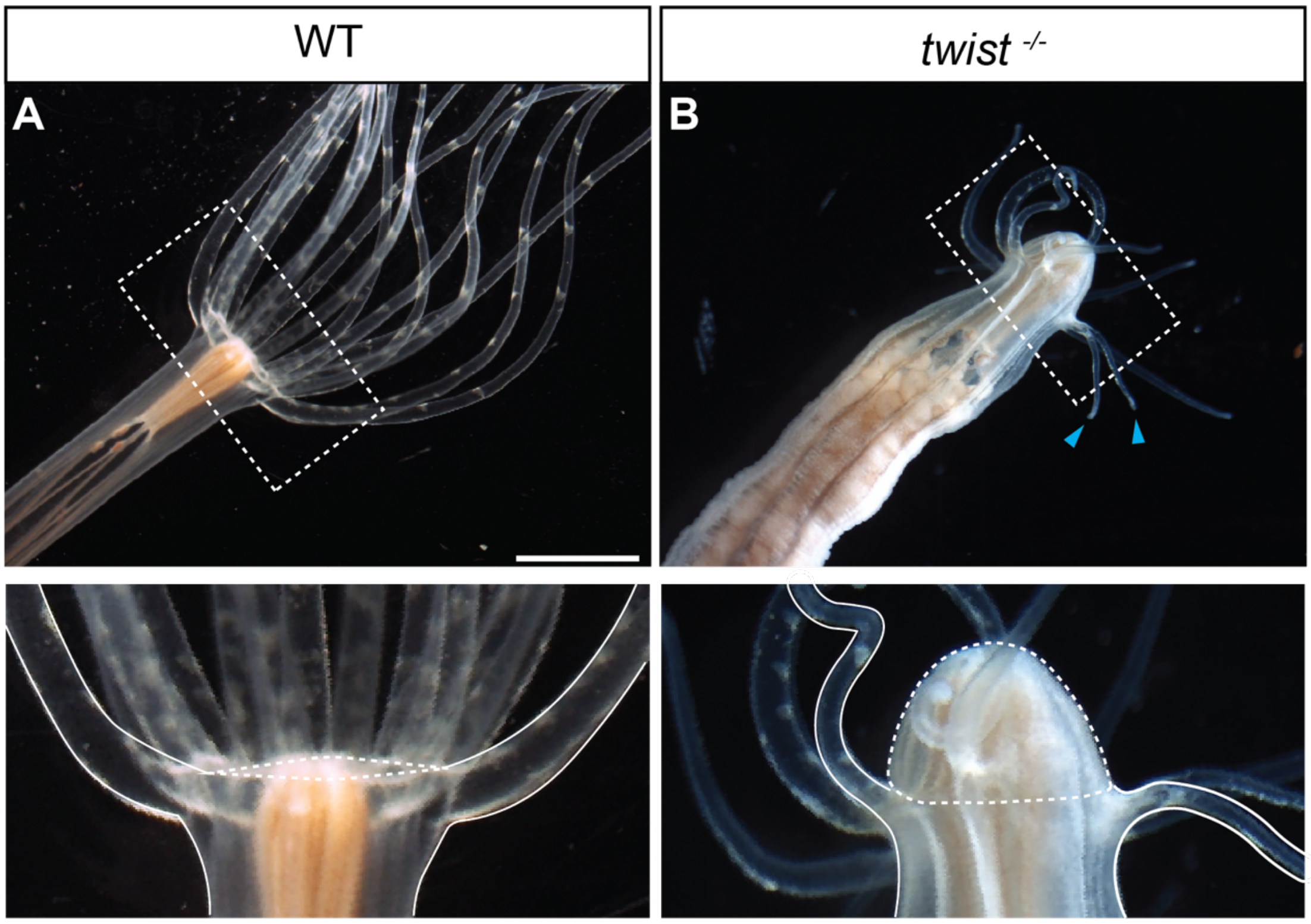

Fig. S3. Adult tentacle number in WT and *twist*$^{-/-}$ animals.

WT polyp displaying typical arrangement of 16 tentacles. **(B)** *twist*$^{-/-}$ mutant at the same developmental stage with reduced tentacle numbers. Lower panels show magnified views of the dashed boxes in (A) and (B), highlighting oral morphology. In *twist-/-* mutants, malformed tentacles (cyan arrowheads) and a distinct trapezoid-shaped hypostome are visible. Panels show representative animals observed across the course of the study. Scalebar 2 mm

Development • Supplementary information

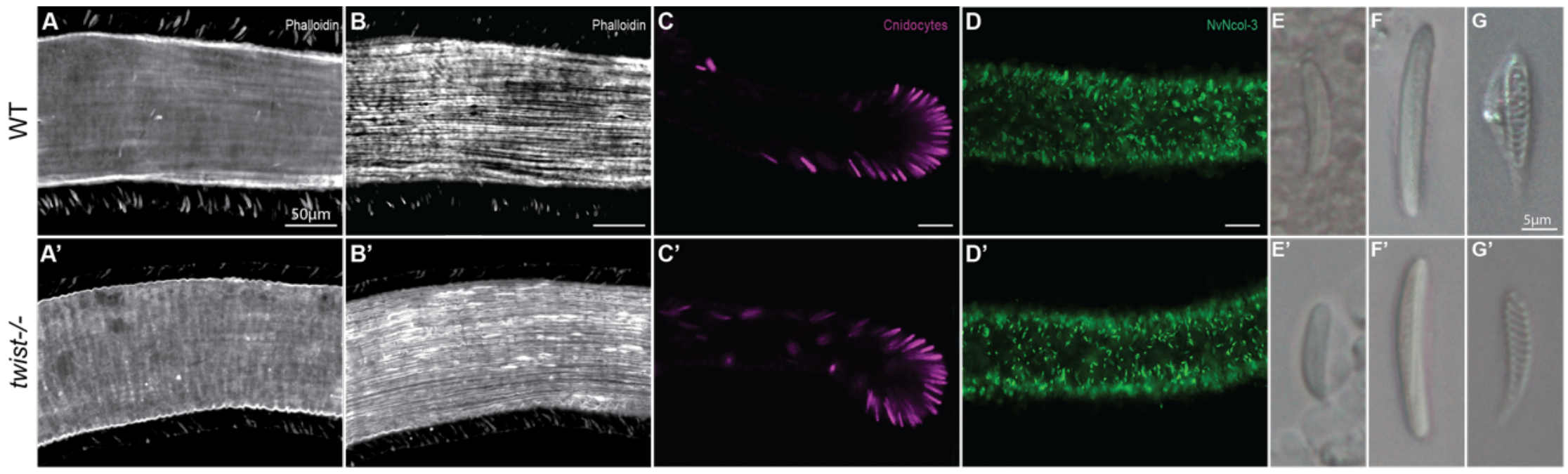

Fig. S4. Muscles and nematocytes of WT and *twist*$^{-/-}$ tentacles.

(A, B) Phalloidin staining of circular mesodermal (A) and ectodermal (B) tentacle muscles in WT animals. Ectodermal (longitudinal) and mesodermal muscles (circular) in *twist*$^{-/-}$ mutants maintain their respective orientations **(A', B')**. **(C, C')** DAPI staining (magenta) of Basitrichous Haplonemas and Microbasic Mastigophores present in both *twist*$^{-/-}$ and WT tentacles. **(D, D')** Immunofluorescence staining of mature cnidocytes Basitrichous Haplonema, Microbasic Mastigophore, and Spirocyst with NvNcol-3 (green) in both *twist*$^{-/}$ and WT tentacles. **(E-G)** DIC microscopy images of Basitrichous Haplonemas, Microbasic Mastigophores, and Spirocysts found in both WT and *twist*$^{-/-}$ mutants **(E'-G')**. n = 6 animals per genotype. Single experiment. All panels show representative individuals. Scale bars are 50µm white and 5µm blue.

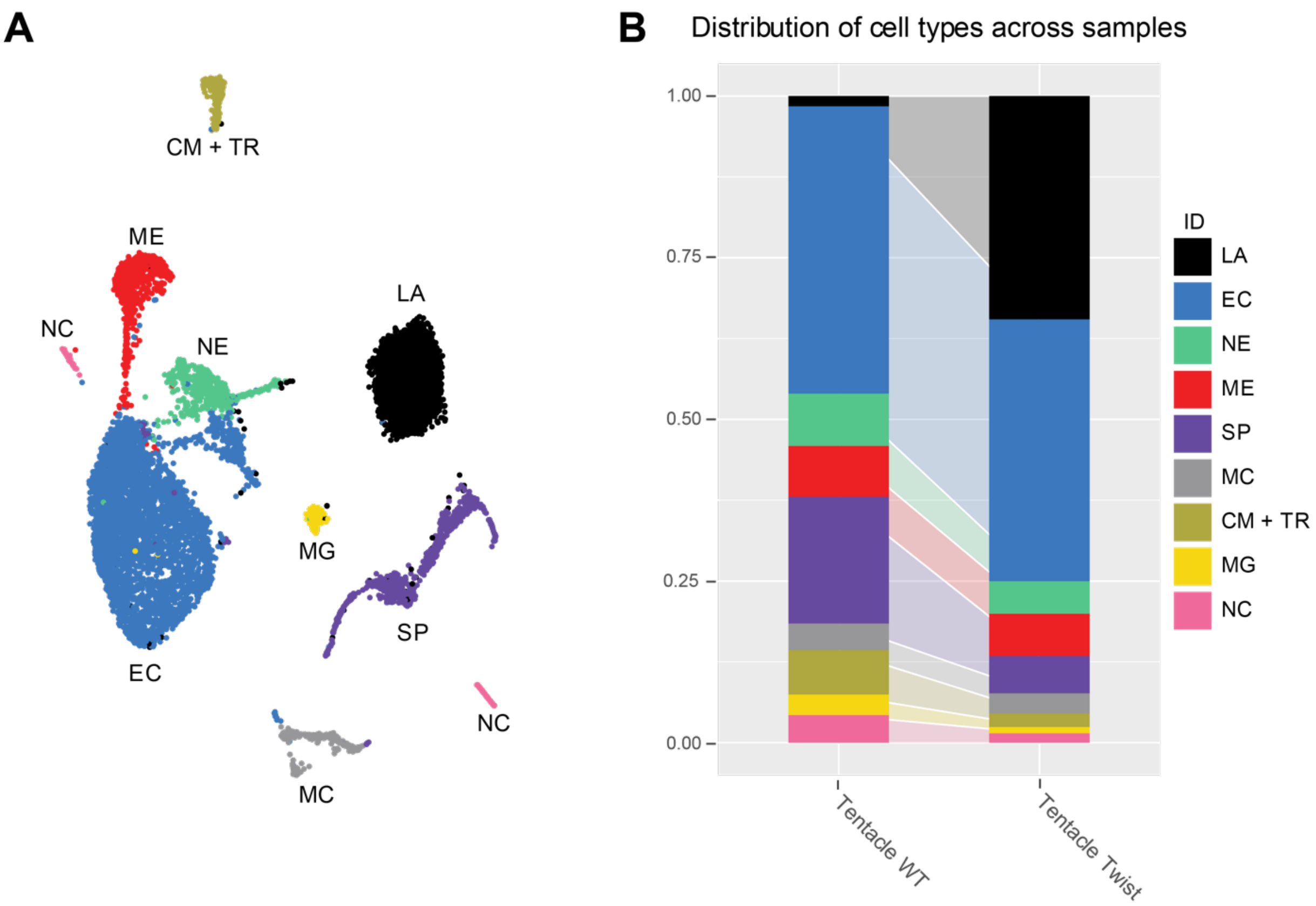

Fig. S5. scRNA-seq Analysis of WT and *twist*$^{-/-}$ tentacle libraries.

(A) Dimplot of both scRNA-seq libraries (Tentacle WT and Tentacle *twist*$^{-/-}$) showing the main clusters found in each library. ME: mesoderm, EC: ectoderm, MG: mucus glands, CN: cnidocytes, MC: mature cnidocytes, SP: spirocytes, CR: circular muscle, TR: tentacle retractor muscle, NE: neurons, NC: not categorized, LA: library artifact. **(B)** Cell type distribution across all samples, showing no absence of characterized clusters but lower ratio of neurons, spirocytes and muscle cell populations in the *twist*$^{-/-}$ library. Data from the same scRNA-seq libraries described in Fig. 10. Each library was generated from a single individual.

Development • Supplementary information

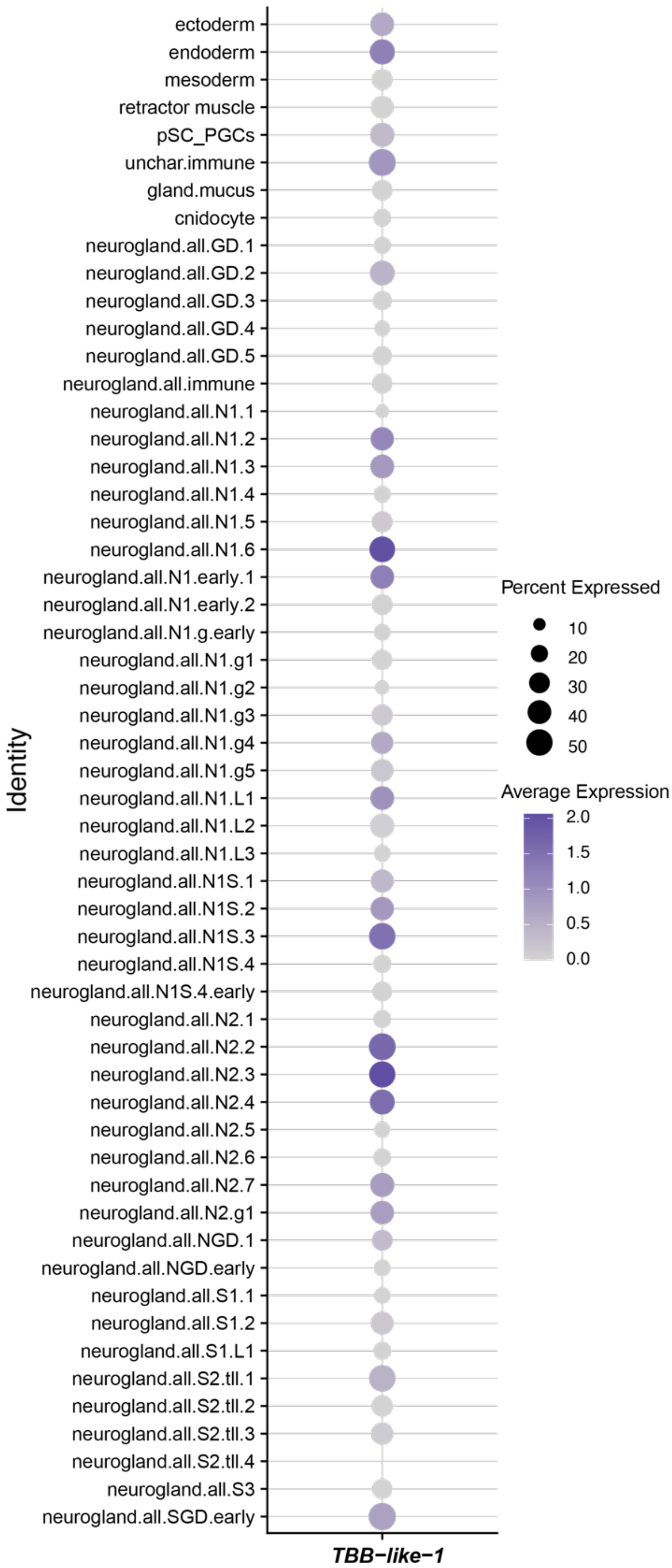

Fig. S6. *tbb-like1* is broadly expressed with enrichment in specific neural subtypes

Dot plot showing expression of *tbb-like1* across all annotated cell clusters in the integrated scRNA-seq dataset. Dot size represents the percentage of cells expressing the gene within each cluster; colour intensity reflects average expression level. While *tbb-like1* is detected in nearly all clusters, moderate enrichment is observed in a subset of neural subtypes. Data from the same scRNA-seq libraries described in Fig. 10. Each library was generated from a single individual.

Table S1. This table contains the full list of Differentially expressed genes derived from bulk RNAseq between twist-/- mutant neoplasm tissue versus wildtype body wall tissue. See also Fig. 7 for a selection of DEGs.

Available for download at
https://journals.biologists.com/dev/article-lookup/doi/10.1242/dev.205340#supplementary-data

Table S2. This table contains the list of differentially expressed transcription factor genes from bulk RNAseq between twist-/- mutant neoplasm tissue versus wildtype body wall tissue. See also Fig. 7.

Available for download at
https://journals.biologists.com/dev/article-lookup/doi/10.1242/dev.205340#supplementary-data

Table S3. This table contains the list of differentially expressed genes coding for tumor markers and oncogenes from scRNAseq between twist-/- mutant neoplasm tissue versus wildtype parietal body wall tissue and tentacle tissue. See also Fig. 7.

Available for download at
https://journals.biologists.com/dev/article-lookup/doi/10.1242/dev.205340#supplementary-data

Table S4. This table contains an overview of all sequencing datasets used in this study with corresponding accession numbers.

Available for download at
https://journals.biologists.com/dev/article-lookup/doi/10.1242/dev.205340#supplementary-data

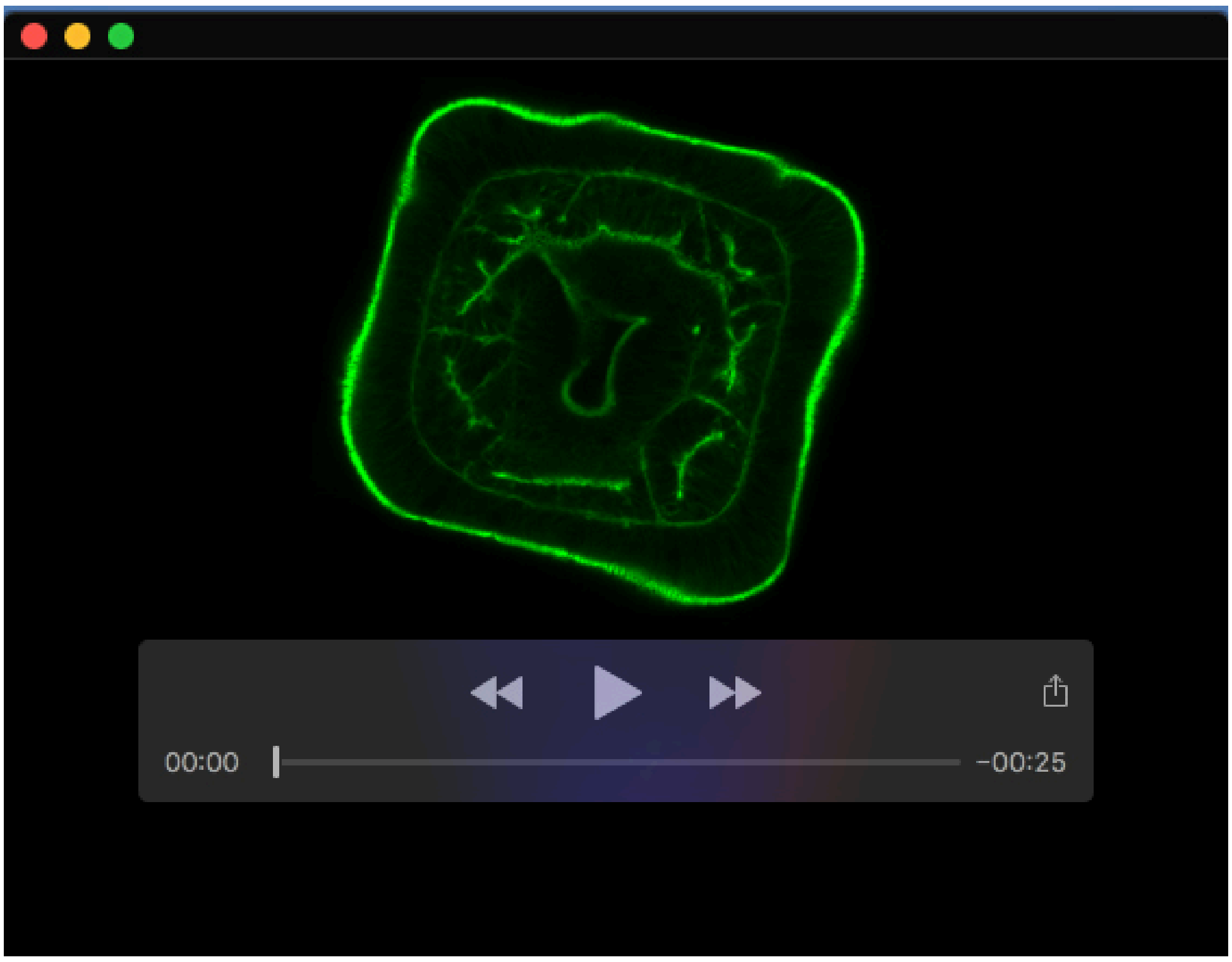

Movie 1. 3D confocal scan (oral view) of the pharynx region of a wildtype animal showing the 8 mesenterial chambers surrounding the pharynx.

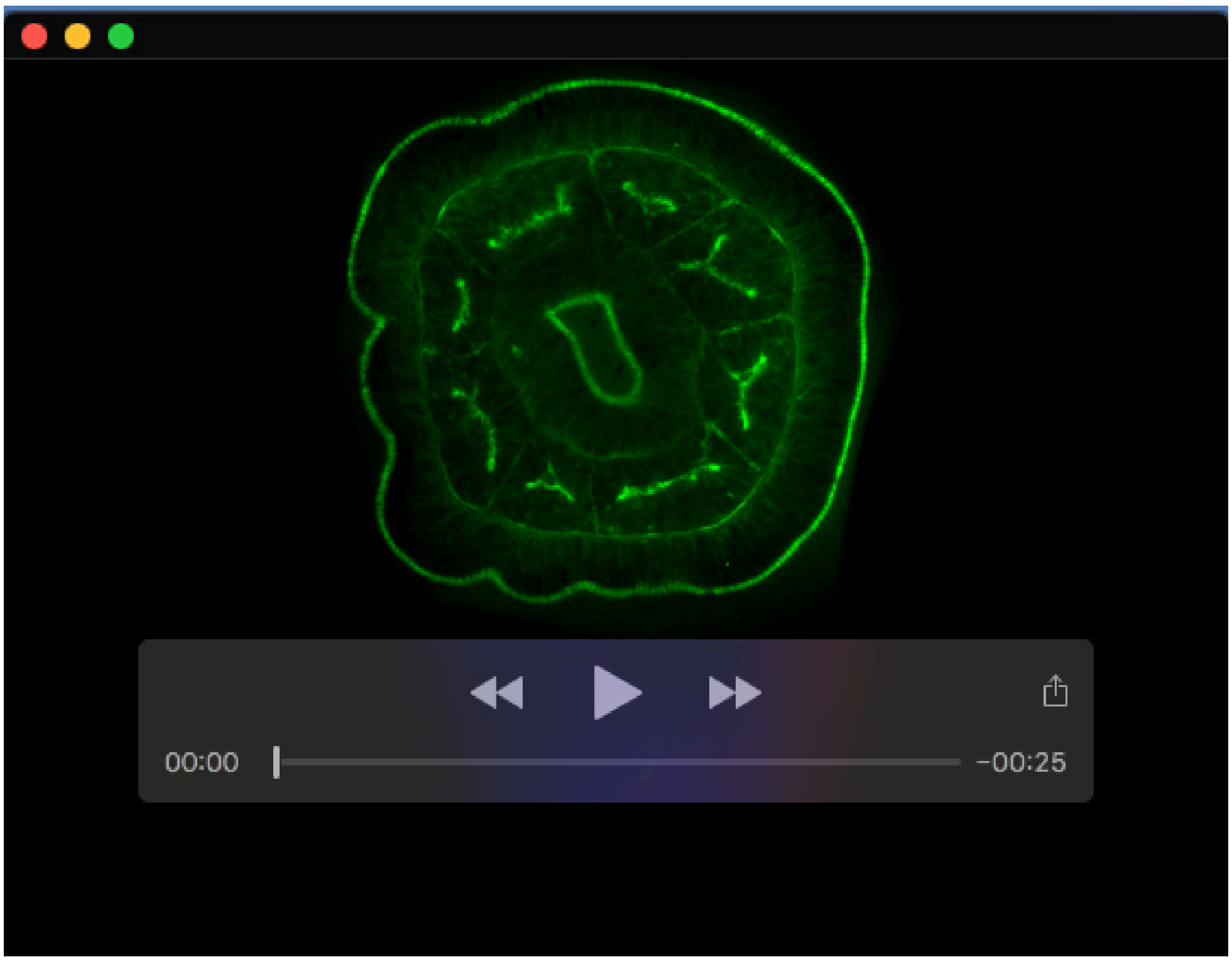

Movie 2. 3D confocal scan (oral view) of the pharynx region of a twist-/- mutant showing that the 8 mesenterial chambers surrounding the pharynx are not affected.

Development • Supplementary information
