# Supplementary material for: Loss of Twist impairs tentacle development and induces epithelial neoplasia in the sea anemone Nematostella vectensis
Source: Development. 2026 Aug 20;153(17):dev205340. doi: 10.1242/dev.205340 (PMC13539428; doi:10.1242/dev.205340)
Supplement: Supplementary file 2 [file develop-153-205340_review_history.pdf]

Loss of Twist impairs tentacle development and induces epithelial neoplasia in the sea anemone *Nematostella vectensis*

Patricio Ferrer Murguia, Julia Hagauer, Emmanuel Haillot, Aissam Ikmi, Juan D. Montenegro, Alison G. Cole and Ulrich Technau
DOI: 10.1242/dev.205340

Editor: Cassandra Extavour

Review timeline
Original submission: 6 November 2025
Editorial decision: 1 January 2026
First revision received: 28 May 2026
Accepted: 7 July 2026

Original submission

First decision letter

MS ID#: dev.205340

MS TITLE: Loss of Twist impairs tentacle development and induces epithelial neoplasia in the sea anemone Nematostella vectensis

AUTHORS: Patricio Ferrer Murguia; Julia Hagauer; Emmanuel Haillot; Aissam Ikmi; Alison G. Cole; Juan Montenegro; Ulrich Technau

Dear Dr Technau,

I have now received all the referees reports on the above manuscript, and have reached a decision. The referees' comments are appended below.

The overall evaluation is positive and we would like to publish a revised manuscript in Development, provided that the referees' comments can be satisfactorily addressed. Please attend to all of the reviewers' comments in your revised manuscript and detail them in your point-by-point response. If you do not agree with any of their criticisms or suggestions explain clearly why this is so. If it would be helpful, you are welcome to contact us to discuss your revision in greater detail. Please send us a point-by-point response indicating your plans for addressing the referees' comments, and we will look over this and provide further guidance.

Reviewer 1

SUMMARY OF THE ADVANCE MADE IN THIS PAPER AND ITS POTENTIAL SIGNIFICANCE TO THE FIELD

This manuscript investigates the regulation of tentacle development in the sea anemone Nematostella vectensis. As relatively little is known about the regulation of appendage development outside arthropods and vertebrates studying tentacle development in this cnidarian provides an important perspective on the evolution of mechanisms underlying appendage development more generally.

© 2026. Published by The Company of Biologists under the terms of the Creative Commons Attribution License (https://creativecommons.org/licenses/by/4.0/).

twist represented an excellent candidate gene for an upstream role in tentacle development so the authors first investigated the expression of this gene further and the phenotypes of twist mutants generated by CRISPR/Cas9. They found that twi mutants has shorter tentacles than wild type supporting a role for this gene in tentacle development. They also showed that twi is required for the formation of the secondary tentacles, which develop later as well as the micronemes. The defective tentacle development is also observed during regeneration evidencing that twi is also required for this process.

To understand where twi sits in regulation of tentacle development they showed that twi expression requires Wnt, BMP and Notch but not MAPK or Hh signalling. Furthermore twi itself is required for expression of paraxis and tbx15 in the pharyngeal mesoderm.

The authors also demonstrated further insights into the underlying mechanisms - showing that two was required for up-regulation of cell proliferation preceding growth of secondary tentacles and correct regulation of TOR activity and the FGF pathway.

The manuscript shows that twi is not required for tentacle muscle development and all cell types appear to be present in mutant tentacles compared to wildtype.

Finally the manuscript demonstrates that the neoplasm-like structures in twi mutants are caused by epithelial hyper proliferation as shown by differentiation of a tentacle like gene expression profile.

Overall this well-written manuscript reports very interesting findings that help better understand the evolution of twi function across animals. The paper shows that twi is required for tentacle development in this cnidarian and places this transcription factor in this gene regulatory network. Interestingly this at least resembles some of the regulatory interactions in vertebrate appendage formation and therefore provides potential evolutionary insights into the genes and interaction involved in the development of appendages more broadly.

SUGGESTIONS TO AUTHORS

It would be helpful if the authors could add a schematic of cnidarian tentacle development to the introduction to support the description in the text for non-specialist readers.

Figure 1. Please sample sizes for F to the figure legend. There is an effect on length but not width clearly but plotting length/width doesn't add anything so could be removed.

Reviewer 2

This manuscript provides an in-depth analysis of the function of Twist in the sea anemone Nematostella vectensis from embryonic development to the adult stage. By generating homozygous CRISPR mutants for Twist, the authors show that post-embryonic mutants do not develop the correct number of tentacles, exhibit an excessively developed hypostome, and form neoplasm-like outgrowths in the oral region that can prevent feeding. The authors combine transgenesis, multiple staining approaches, inhibitor treatments, and single-cell RNA sequencing. They demonstrate that Twist is regulated by BMP and Wnt signaling pathways during embryogenesis, but likely not by the MAPK and Hedgehog pathways. They further show that Twist is required for secondary tentacle formation, likely through interactions with the FGF and TOR signaling pathways. Finally, they characterize the outgrowths observed in Twist mutants and show that these 'neoplasms' display tentacle-specific features, suggesting that Twist is necessary for maintaining tissue homeostasis at the oral pole. Altogether, this study represents a highly valuable contribution to our understanding of tentacle development in Nematostella. However, I have several comments that I would like to see addressed before publication.

Major points to be addressed:

1) The authors compared the phenotype of wild-type and Twist homozygous mutants at the planula and primary polyp stages and did not identify any clear differences. They argue that the function of Twist becomes evident only during secondary tentacle growth. For instance, the authors wrote "In

© 2026. Published by The Company of Biologists under the terms of the Creative Commons Attribution License (https://creativecommons.org/licenses/by/4.0/).

unfed polyps, both WT and twist-/- mutants initially exhibited similar levels of detectable Fgfrb::eGFP positive cells (Fig. 5A', D')." Comparing Figures 5A' and 5D', the level of detectable Fgfrb::eGFP seems to me reduced in Twist-/- mutants. Similarly, the shape of the pharynx at the primary polyp stage (according to Figures 1B, C), as well as the shape of the mesenteries (when comparing Figure S2), seem different. I would suggest expanding the measurements performed on WT vs Twist-/- planula and primary polyps beyond the overly simple body length/width, tentacle length/width to better quantify the actual differences between WT and mutants before the first feeding. It could also be interesting to assess the impact on gene expression of the Twist knockout beyond Paraxis and Tbx15.

2) One important missing piece of information for understanding the Twist-/- mutant phenotype is the pattern of Twist expression in post-feeding polyps. This could be assessed by in situ hybridization.

3) Using scRNA-seq, the authors show that the neoplasm-like structures, which form in Twist-/- mutants, resemble tentacle tissue in terms of cell types. Given that Twist is involved in many cancers in vertebrates, it would be insightful to assess whether these neoplasm cells overexpress cancer-related genes. Such an analysis would provide an interesting comparison with other systems and help validate their designation as neoplasms.

4) Related to the previous comment, it is not clear to me how neoplasms start forming. In Figure 8B, we can see that one mesentery is missing at the location of the neoplasm. Is this a common observation? Do gastrodermal cells proliferate in those structures together with ectodermal cells? Do mesenteries play any role in their early formation?

5) From the information provided in the Results and Methods sections, it is not clear which scRNA-seq samples were produced during this study and which ones were reused from previous studies. Similarly, the Data Availability explicitly mentions "Twist KO tentacle" and "Twist KO neoplasm" but not the other newly generated sequencing datasets. It would be helpful to detail in the Methods the different libraries generated and clearly provide their individual accessions in the Data Availability section.

6) Figure S5 presents a comparative analysis of WT and Twist-/- scRNA-seq tentacle libraries. Related to this, the authors state: "When comparing the relative abundance of each cell type, normalized to the total of cells per sample, we observed a decrease in spirocytes, muscle cells, neurons and mucus glands." However, a large portion of cells in the "tentacle Twist-/-" sample correspond to a library artefact (LA). How did the authors normalize their samples? Why did they keep those artefact cells? Does removing them impact their conclusions?

7) The Discussion section is very long, quite speculative, and feels more like a review on the function of Twist in animals. I would suggest reducing it (by at least a third), focusing on the key findings of this study.

Minor details:

8) Please replace "microneme" by the correct anatomical term "microcneme" throughout the manuscript.

9) Missing bracket on page 6 "the hypostome of hydrozoans (Fig 1C, E')"'

10) Figure 1 legend. There seems to be an issue with (F), "(left)" instead of (top) and "(right)" instead of (middle).

11) Figure legends 3C, 4, 5. Please indicate how old the polyps are.

First revision

© 2026. Published by The Company of Biologists under the terms of the Creative Commons Attribution License (https://creativecommons.org/licenses/by/4.0/).

Author response to reviewers' comments

Reviewer 1 – Comment 1

It would be helpful if the authors could add a schematic of cnidarian tentacle development to the introduction to support the description in the text for non-specialist readers.

Response:

We thank the reviewer for this helpful suggestion. To facilitate understanding of tentacle development for non-specialist readers, we have added a schematic overview of *Nematostella* tentacle development illustrating primary and secondary tentacle formation, mesenteries, and the appearance of microcnemes. This schematic is now included as **Figure S1** and referenced in the Introduction.

Reviewer 1 – Comment 2

Figure 1. Please add sample sizes for F to the figure legend. There is an effect on length but not width clearly but plotting length/width doesn't add anything so could be removed.

Response:

We thank the reviewer for this constructive suggestion. Sample sizes have now been added to the Figure 1F legend. In addition, we have removed the length/width ratio plot, as it did not provide additional information beyond the absolute tentacle length measurements.

Reviewer 2 – Major Comment 1

The authors compared the phenotype of wild-type and Twist homozygous mutants at the planula and primary polyp stages and did not identify any clear differences. They argue that the function of Twist becomes evident only during secondary tentacle growth. For instance, the authors wrote "In unfed polyps, both WT and twist-/- mutants initially exhibited similar levels of detectable Fgfrb::eGFP positive cells (Fig. 5A', D')." Comparing Figures 5A' and 5D', the level of detectable Fgfrb::eGFP seems to me reduced in Twist-/- mutants. Similarly, the shape of the pharynx at the primary polyp stage (according to Figures 1B, C), as well as the shape of the mesenteries (when comparing Figure S2), seem different. I would suggest expanding the measurements performed on WT vs Twist-/- planula and primary polyps beyond the overly simple body length/width, tentacle length/width to better quantify the actual differences between WT and mutants before the first feeding. It could also be interesting to assess the impact on gene expression of the Twist knockout beyond Paraxis and Tbx15.

Response:

We thank the reviewer for this careful and insightful comment. We agree that subtle morphological and quantitative differences between WT and *twist*$^{-/-}$ animals can already be observed at the primary polyp stage, and we have revised the manuscript to clarify this point and avoid overstatement.

Regarding FGFRb::eGFP expression in unfed primary polyps, we acknowledge that in individual representative images the signal can appear reduced in *twist*$^{-/-}$ animals. However, this impression can be influenced by differences in morphology and imaging plane in individual images. At early, unfed primary polyp stages, FGFRb::eGFP-positive cells were consistently detectable in both WT and *twist*$^{-/-}$ animals, and apparent differences in signal intensity were subtle and variable between individuals. Given the inherent variability of whole-mount fluorescence intensity at this stage, we did not consider quantitative fluorescence measurements to be sufficiently robust. In contrast, at later stages during secondary tentacle development, FGFRb::eGFP signal reduction in *twist*$^{-/-}$ animals is pronounced and reproducible across animals, and is therefore described qualitatively in the manuscript. We have adjusted the wording in the manuscript to reflect this distinction.

© 2026. Published by The Company of Biologists under the terms of the Creative Commons Attribution License (https://creativecommons.org/licenses/by/4.0/).

With respect to early morphological differences, we confirm that alterations, albeit subtle, are already visible at the primary polyp stage. As described in the Results section, these differences primarily reflect changes in oral disc morphology, resulting in a hypostome-like protrusion, rather than defects in pharynx formation per se. Consistent with this, we do not observe gross alterations in pharynx structure at this stage. We have clarified this point in the text to ensure correct anatomical interpretation.

The apparent differences in mesenteries noted in Supplementary Fig. S2 were due to the choice of sagittal plane shown in the representative still image. Examination of the corresponding movie data and additional animals indicates that mesentery number and overall organization are comparable between WT and *twist-/-* polyps at this stage. To avoid confusion, we have replaced the still image with a more representative view.

Finally, we agree that body length/width measurements alone provide only a coarse assessment of early developmental phenotypes. We have therefore softened our wording throughout the manuscript to state that *twist*$^{-/-}$ animals do not exhibit gross morphological defects at early stages, while modest quantitative and morphological differences are already detectable prior to secondary tentacle growth.

Regarding gene expression, we selected *paraxis* and *tbx15* as representative mesodermal transcription factors based on their overlapping expression domains with *twist* and their relevance to mesodermal differentiation. To assess the broader effect of Twist on downstream transcriptional program, we carried out a bulk RNAseq (with three replicates) of wildtype and mutant heads in juvenile polyps and determined the upregulated and downregulated genes in a DEG analysis. This analysis confirmed the downregulation of *paraxis* and *tbx15*, but also revealed the differential expression of several other transcription factors and signaling pathways. We show the new data in the new Figure 7 and the full dataset in Suppl. Table 1 and 2.

Reviewer 2 – Major Comment 2

One important missing piece of information for understanding the Twist-/- mutant phenotype is the pattern of Twist expression in post-feeding polyps. This could be assessed by in situ hybridization.

Response:

In situ hybridization in juvenile or adult polyps is often notoriously difficult and prone to background. However, as mentioned above, we have examined *twist* expression in post-feeding polyps by bulk RNAseq in both wildtype and mutant and find that *twist* remains detectable in the oral region at these stages (Fig. 7; Supplementary Table 1, 2).

Reviewer 2 – Major Comment 3

Using scRNA-seq, the authors show that the neoplasm-like structures, which form in Twist$^{-/-}$ *mutants, resemble tentacle tissue in terms of cell types. Given that Twist is involved in many cancers in vertebrates, it would be insightful to assess whether these neoplasm cells overexpress cancer-related genes. Such an analysis would provide an interesting comparison with other systems and help validate their designation as neoplasms.*

Response:

We thank the reviewer for this thoughtful suggestion. We examined the differential gene expression profile of the neoplasm bulk RNAseq in comparison to known tumor-associated genes. As detailed in the manuscript and Supplementary Table 1, we identified upregulation of *tbb-like1* and *TCTP-like-1*, both of which have been implicated in tumorigenesis in other systems. However, we did not detect a broad activation of canonical oncogenic transcriptional programs or markers of malignant transformation.

Together with our EdU data demonstrating elevated proliferation, these findings support the interpretation that the structure represents a hyperproliferative epithelial neoplasm-like outgrowth

© 2026. Published by The Company of Biologists under the terms of the Creative Commons Attribution License (https://creativecommons.org/licenses/by/4.0/).

with tentacle-like profiles rather than a fully transformed malignant tissue. This is why we call this a neoplasm and not a cancer. We have clarified this distinction in the revised manuscript to avoid overinterpretation.

Reviewer 2 – Major Comment 4

Related to the previous comment, it is not clear to me how neoplasms start forming. In Figure 8B, we can see that one mesentery is missing at the location of the neoplasm. Is this a common observation? Do gastrodermal cells proliferate in those structures together with ectodermal cells? Do mesenteries play any role in their early formation?

Response:

We thank the reviewer for this thoughtful question regarding the origin of the neoplasm-like structures. The apparent absence of a mesentery at the site of the neoplasm in the original representative image reflected the specific orientation and section shown. Examination of several additional animals and sections indicates that neoplasms are observed in the presence of the full complement of mesenteries, demonstrating that mesentery loss is not a consistent or defining feature of these structures. We have replaced the image with a more representative view to avoid confusion.

Morphologically, the neoplasms appear as localized hyperplastic expansions of both epithelial layers at the level of the pharynx, while preserving overall tissue organization and radial architecture. The expansion always starts between two mesenteries. Consistent with this interpretation, our single-cell RNA-seq data indicate that the neoplasm exhibits a mixed transcriptional identity: it shares features with tentacle tissue but does not fully recapitulate a canonical tentacle profile, nor does it correspond entirely to body wall identity.

While EdU labeling demonstrates elevated proliferation within these structures, our current data do not allow us to resolve the precise earliest stages of neoplasm initiation or to definitively distinguish the relative contributions of ectodermal and gastrodermal cells. At present, we do not have evidence that mesenteries play a causal role in neoplasm formation, and we have clarified this point in the manuscript to avoid speculative interpretation.

Reviewer 2 - Major comment 5

It is not clear which scRNA-seq samples were produced during this study and which ones were reused from previous studies. Similarly, the Data Availability explicitly mentions "Twist KO tentacle" and "Twist KO neoplasm" but not the other newly generated sequencing datasets. It would be helpful to detail in the Methods the different libraries generated and clearly provide their individual accessions in the Data Availability section.

Response:

We thank the reviewer for pointing this out. All scRNA-seq libraries used for the neoplasm analysis (tentacle WT, tentacle twist-/-, interparietal WT, and neoplasm) were newly generated for this study and are available under GEO accession GSE308593. Previously published datasets were only used for comparative analyses and are listed separately in the Data Availability section and Supplementary Table 3. We have clarified this in the Methods section.

Reviewer 2 - Major comment 6

A large portion of cells in the "tentacle Twist$^{-/-}$" sample correspond to a library artefact (LA).How did the authors normalize their samples?

Why did they keep those artefact cells?

Does removing them impact their conclusions?

© 2026. Published by The Company of Biologists under the terms of the Creative Commons Attribution License (https://creativecommons.org/licenses/by/4.0/).

Response:

We thank the reviewer for raising this important point. The “LA” cluster represents a likely dissociation/ambient RNA artefact. It is almost exclusively present in tentacle_twist samples, which were fairly difficult to dissociate, and it displays overlapping marker expression from multiple otherwise distinct cell types without a coherent lineage-defining transcriptional program, consistent with barcode contamination or lysed-cell transcript capture.

In the original analysis, relative cell-type proportions were calculated using all cells per sample. To assess robustness, we recalculated proportions after excluding the LA cluster from the denominator and normalizing only to non-LA biological cells. The LA cluster constituted ~4.3% of tentacle_twist cells and ~0.13% of tentacle_wt cells. Exclusion of this population resulted in only minor proportional rescaling (≤0.7% absolute change per cluster) and did not alter any relative enrichment trends or biological conclusions.

We have clarified this normalization procedure in the Methods section and now explicitly state that composition analyses are robust to exclusion of the LA cluster.

Reviewer 2 – Major Comment 7

The Discussion section is very long, quite speculative, and feels more like a review on the function of Twist in animals. I would suggest reducing it (by at least a third), focusing on the key findings of this study.

Response:

We thank the reviewer for this helpful suggestion. We have completely revised and shortened the Discussion section, focusing on the key findings of this study and streamlining speculative evolutionary comparisons. Sections discussing broader Twist functions in other metazoans have been condensed to better emphasize the central conclusions.

Reviewer 2 – Major Comment 8

Please replace "microneme" by the correct anatomical term "microcneme" throughout the manuscript.

Response:

We thank the reviewer for pointing this out. We have replaced all instances of “microneme” with the correct anatomical term “microcneme” throughout the manuscript.

Reviewer 2 – Major Comment 9

Missing bracket on page 6 "the hypostome of hydrozoans (Fig 1C, E')"'

Response:

The missing bracket has been corrected.

Reviewer 2 – Major Comment 10

Figure 1 legend. There seems to be an issue with (F), "(left)" instead of (top) and "(right)" instead of (middle).

Response:

The panel description in the Figure 1 legend has been corrected to accurately reflect the layout of the boxplots.

Reviewer 2 – Major Comment 11

© 2026. Published by The Company of Biologists under the terms of the Creative Commons Attribution License (https://creativecommons.org/licenses/by/4.0/).

Figure legends 3C, 4, 5. Please indicate how old the polyps are.

Response:

We have now specified the developmental stage/age of the polyps in the legends of Figures 3C, 4, and 5 to improve clarity.

Second decision letter

MS ID#: dev.205340R1

MS TITLE: Loss of Twist impairs tentacle development and induces epithelial neoplasia in the sea anemone Nematostella vectensis

AUTHORS: Patricio Ferrer Murguia; Julia Hagauer; Emmanuel Haillot; Aissam Ikmi; Alison G. Cole; Juan Montenegro; Ulrich Technau

Dear Dr Technau,

I am happy to tell you that your manuscript has been accepted for publication in Development, pending our standard publication integrity checks. It was accepted on 07 Jul 2026.

Reviewer 1

SUMMARY OF THE ADVANCE MADE IN THIS PAPER AND ITS POTENTIAL SIGNIFICANCE TO THE FIELD

This paper describes interesting data on the role of twist in tentacle formation in Nematostella. Given the interactions uncovered by the study their work provides important insights into the evolution and development of animal appendages more broadly.

SUGGESTIONS TO AUTHORS

The authors have rigorously addressed all of my comments and those of the other reviewer, and added new data to the manuscript to support their interesting findings.

Reviewer 2

The authors have appropriately addressed my comments, and I am pleased to recommend that this manuscript be accepted for publication.

© 2026. Published by The Company of Biologists under the terms of the Creative Commons Attribution License (https://creativecommons.org/licenses/by/4.0/).
